# Human in vivo-generated monocyte-derived dendritic cells and macrophages cross-present antigens through a vacuolar pathway

**DOI:** 10.1038/s41467-018-04985-0

**Published:** 2018-07-02

**Authors:** Tsing-Lee Tang-Huau, Paul Gueguen, Christel Goudot, Mélanie Durand, Mylène Bohec, Sylvain Baulande, Benoit Pasquier, Sebastian Amigorena, Elodie Segura

**Affiliations:** 1 Institut Curie, PSL Research University, INSERM, U932, 26 rue d’Ulm, 75005 Paris, France; 2 Sanofi, Breakthrough Laboratory, 1 Impasse des Ateliers, 94400 Vitry-sur-Seine, France; 3 Institut Curie, PSL Research University, NGS Platform, 26 rue d’Ulm, 75005 Paris, France

## Abstract

Presentation of exogenous antigens on MHC-I molecules, termed cross-presentation, is essential for cytotoxic CD8^+^ T cell responses. In mice, dendritic cells (DCs) that arise from monocytes (mo-DCs) during inflammation have a key function in these responses by cross-presenting antigens locally in peripheral tissues. Whether human naturally-occurring mo-DCs can cross-present is unknown. Here, we use human mo-DCs and macrophages directly purified from ascites to address this question. Single-cell RNA-seq data show that ascites CD1c^+^ DCs contain exclusively monocyte-derived cells. Both ascites mo-DCs and monocyte-derived macrophages cross-present efficiently, but are inefficient for transferring exogenous proteins into their cytosol. Inhibition of cysteine proteases, but not of proteasome, abolishes cross-presentation in these cells. We conclude that human monocyte-derived cells cross-present exclusively using a vacuolar pathway. Finally, only ascites mo-DCs provide co-stimulatory signals to induce effector cytotoxic CD8^+^ T cells. Our findings thus provide important insights on how to harness cross-presentation for therapeutic purposes.

## Abstract (web summary)

Cross-presentation, or the presentation of exogenous antigens on MHC-I, is thought to be restricted to dendritic cells (DCs). Here the authors show that human DCs and macrophages developed in vivo from monocytes can both perform cross-presentation using a non-conventional pathway, but only DCs are capable of inducing cytotoxic CD8^+^ T cells.

## Introduction

Cross-presentation is essential for the induction of cytotoxic CD8^+^ T cells and efficient immune responses against infections or cancer^1^. Numerous studies in mice have shown that cross-presentation is performed by dendritic cells (DCs). DCs can be classified into four subsets based on ontogeny^2^. “Classical” Batf3-dependent DC1 (cDC1), “classical” Batf3-independent DC2 (cDC2), and plasmacytoid DCs (pDCs) derive from pre-committed bone marrow precursors. Monocyte-derived DCs (mo-DCs) arise from monocytes recruited into tissues and become the most abundant DC population during inflammation. In mice, cross-presentation is mainly performed by cDC1 in lymphoid organs^1,3^, but mo-DCs have the unique ability to cross-present antigens to CD8^+^ T cells directly in peripheral tissues^4–6^. Cross-presentation by mo-DCs has a crucial role in the rapid activation of tissue-resident memory CD8^+^ T cells upon infection^4^ and in the efficacy of anti-tumoral treatments based on immunostimulatory agents or chemotherapy^5,7^. Harnessing the cross-presentation capacity of mo-DCs for therapeutic intervention is therefore an attractive prospect. However, determining whether human mo-DCs that arise in tissues can cross-present, and the molecular mechanisms involved, will be a prerequisite.

We and others have shown that the functional specialization for cross-presentation is not conserved between mouse and human DC subsets. In contrast to mouse DCs, human cDC1, cDC2, and pDCs all have a similar ability to cross-present antigens^8–11^. Human mo-DCs generated in vitro from monocytes cultured with GM-CSF and IL-4 can cross-present, and have long been used as a model to understand the biology of cross-presentation, however this culture system gives rise to DCs that do not closely resemble naturally-occurring mo-DCs found in vivo in inflammatory fluids^12^. Therefore, the cross-presentation ability of human mo-DCs remains unclear.

Here, we address this question using human in vivo-generated mo-DCs, directly isolated from peritoneal ascites from cancer patients^12,13^. We find that mo-DCs and monocyte-derived macrophages (mo-Mac) can both cross-present efficiently, using exclusively a vacuolar pathway. However, only mo-DCs are able to produce co-stimulatory signals for the induction of effector cytotoxic CD8^+^ T cells.

## Results

### Tumor ascites CD1c^+^ DCs are monocyte-derived cells

**Fig. 1:**
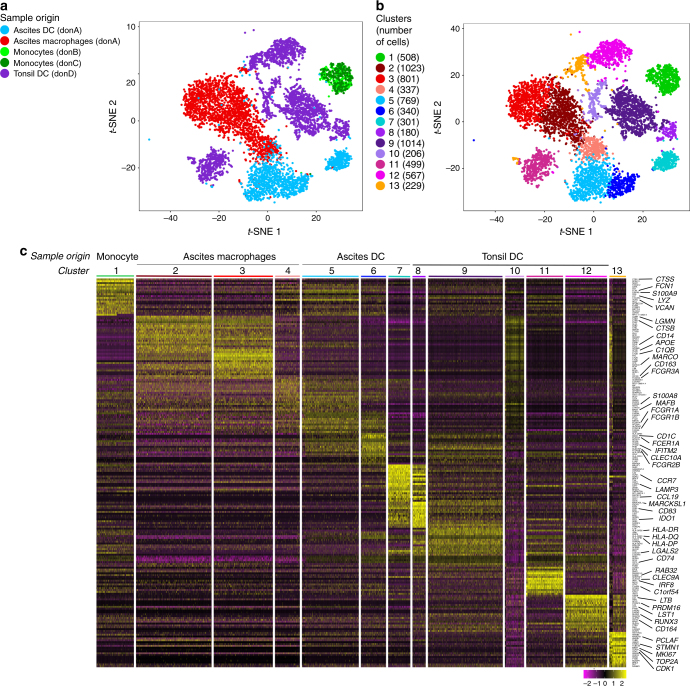
Ascites DCs are distinct from classical DCs. Purified ascites DCs, ascites macrophages, tonsil DCs, and blood monocytes were analyzed by single-cell RNA-seq using a Drop-seq approach. Combined single-cell transcriptomes were analyzed. **a**, **b** *t*-SNE representation of cell clusters identified using unsupervised clustering. Each dot represents an individual cell. **a** Colors represent sample origin; don donor. **b** Colors represent identified clusters. Clusters are manually ordered and cell numbers for each cluster is indicated. **c** Heatmap of scaled expression (log values of UMI) for the top 20 differentially expressed genes of each cluster (based on log fold change)

Based on phenotype and gene expression analysis, we have identified the CD1c^+^ DC population found in tumor ascites as naturally-occurring mo-DCs^12,13^. Because of the sensitivity of the functional assay for cross-presentation, a minor population of cDC within ascites DCs could bias our results. Therefore, we first sought to address the heterogeneity of ascites DCs using single-cell RNA-seq analysis. We purified ascites DCs (gated as HLA-DR^+^CD11c^+^CD1c^+^CD16^−^), ascites macrophages (gated as HLA-DR^+^CD11c^+^CD1c^−^CD16^+^) and, for comparison, tonsil cDCs (gated as HLA-DR^+^CD11c^+^CD14^−^), and analyzed single-cell transcriptomes using a droplet-based method enabling 3′ mRNA counting^14^. To increase the power of the analysis, we combined this dataset with that of blood CD14^+^ monocytes that we had previously generated^12^. To evaluate the heterogeneity of these population, we performed unsupervised clustering using a graph-based approach with the Seurat package^15^. For visualization of the cell clusters, we used *t*-distributed stochastic neighbor embedding (*t*-SNE). Unsupervised clustering of the combined dataset identified 13 main clusters (Fig. 1a, b), and three minor clusters of contaminating cells that were removed from subsequent analysis (see Methods for details). Cluster 1 contained cells from monocytes; clusters 2–3: cells from the macrophage sample; cluster 4: cells from both ascites DCs and macrophages samples; clusters 5–7: cells from the ascites DCs sample; clusters 8–12: cells from the tonsil DCs sample; and cluster 13: cells from the ascites DCs, ascites macrophages, and tonsil DCs samples (Fig. 1a,b).

We then analyzed differentially expressed genes between clusters (Fig. 1c and Supplementary Fig. 1). Cluster 1 displayed high expression of monocyte genes such as *CTSS, FCN1, S100A9, LYZ, VCAN*. Clusters 2 and 3 shared high expression of macrophage genes such as *LGMN, CTSB, CD14, APOE, C1QB, MARCO, CD163, FCGR3A*. Cluster 4 expressed high levels of monocyte and macrophage-related genes such as *FCN1, S100A9, VCAN, S100A8, MAFB*. Clusters 5 and 6 expressed monocyte-related genes such as *FCN1, S100A9, VCAN, FCGR1A, FCGR1B*, as well as DC genes including *CD1C, FCER1A, IFITM2, CLEC10A, FCGR2B*. Clusters 7 and 8 showed high expression of DC activation genes *CCR7, LAMP3, CCL19, MARCKSL1, CD83, IDO1*. Cluster 9 expressed cDC2 hallmark genes *CD1C, FCER1A, CLEC10A*. Cluster 10 displayed high expression of macrophage genes *LGMN, CTSB, CD14, APOE, MARCO, CD163, FCGR3A, S100A8, MAFB*. Cluster 11 showed high expression of cDC1 genes such as *RAB32, CLEC9A, IRF8, C1orf54, IDO1*. Cluster 12 had high expression of genes expressed on progenitors or related to cDC development such as *LTB, PRDM16, LST1, RUNX3, CD164*. Finally, cluster 13 showed high levels of cell cycle genes including *PCLAF, STMN1, MKI67, TOP2A, CDK1*. Of note, cluster 13 contained cells from three different samples (ascites DCs, ascites macrophages, and tonsil DCs), showing that in this analysis, cells with similar transcriptional programs are grouped in the same cluster independently of their sample origin.

**Fig. 2:**
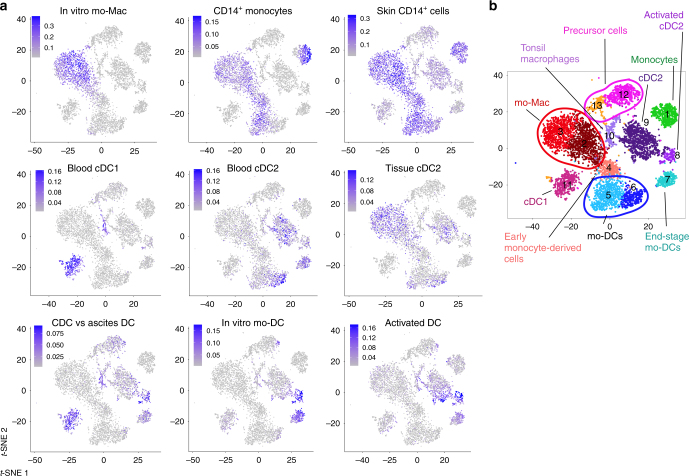
Ascites DCs are monocyte-derived cells. Purified ascites DCs, ascites macrophages, tonsil DCs, and blood monocytes were analyzed by single-cell RNA-seq using a Drop-seq approach. Combined single-cell transcriptomes were analyzed. **a** Signature scores in individual cells for indicated gene signatures. **b** Annotation of cell clusters

To confirm the identity of these clusters, we analyzed signature scores in individual cells for several sets of gene signatures (Fig. 2a). For each cell, we calculated the average expression of each signature, substracted by the aggregated expression of control gene sets^16^. We used published gene signatures for blood cDC1^17^, blood cDC2^17^, CD14^+^ monocytes^17^, and skin CD14^+^ cells^17^ (Supplementary Data 1). We also designed signatures for tissue cDC2 by combining transcriptomic data from blood and spleen cDC2^18^, for in vitro-generated mo-Mac and mo-DCs (obtained with M-CSF, IL-4, and TNFa^12^), for genes enriched in blood cDC2 compared to ascites DCs and ascites macrophages^13^, and for “activated DC” by selecting genes enriched in both blood cDC2 and in vitro-generated mo-DCs (obtained with GM-CSF and IL-4) exposed to the same stimulus (Menomune microbial vaccine)^19^. Complete lists of genes and strategy for each signature are shown in Supplementary Data 1. Only clusters 2 and 3 expressed the in vitro mo-Mac signature, confirming the identity of these cells as macrophages. As expected from its cellular origin, cluster 1 had the highest score for the CD14^+^ monocyte signature, but clusters 2, 3, 4, and 5 also displayed high scores for this signature. In addition, clusters 1–6 had high scores for the signature of skin CD14^+^ cells, which have been shown to derive from monocytes^20^. This is consistent with the notion that ascites DCs and macrophages are related to monocytes. Cluster 11 had the highest score for the blood cDC1 gene signature, confirming the results from differential gene expression. Clusters 6, 7, and 9 had the highest scores for the blood cDC2 signature. However, when using the signature for tissue cDC2, we found that clusters 2, 3, and 9 had the highest scores, with some cells from clusters 5 and 6 also displaying high scores. This suggests that markers for cDC2 may be less robust than for other cell types, possibly due to similar transcriptional programs between cDC2 and other antigen-presenting cells (APC). Consistent with this, when analyzing expression scores for genes enriched in cDC2 compared to ascites DCs and macrophages, we found that clusters 11 and 8 displayed the highest scores. Clusters 7 and 8 had the highest score for the in vitro mo-DC signature, with clusters 9 and 11 displaying low scores. Finally, cluster 8 had the highest score for the “activated DC” signature, with some cells from cluster 9 also displaying high scores, and cluster 7 showing an intermediate score for this signature.

Cluster 7 (from ascites DCs) and cluster 8 (from tonsil DCs) shared a high number of markers genes (Fig. 1c) and displayed mixed gene signatures (Fig. 2a). Nevertheless, these cells are not grouped in the same cluster by the clustering algorithm, independently of the resolution used (Supplementary Fig. 2), suggesting that there are significant differences in their transcriptional profile driving their identification as distinct population. Similar profiles could be explained by the convergence of transcriptional programs of mo-DCs and cDC2, in particular for maturation genes, as observed for activated mouse DCs of distinct ontogeny^21^. Alternatively, the separation into distinct clusters could be driven by differences due to tissue origin (fluid versus lymphoid organ). To directly address whether ascites DCs contain a population of cDCs in cluster 7 or whether tonsil DCs contain a population of mo-DCs in cluster 8, we analyzed genes that are the most differentially expressed between cluster 7 (containing ascites DCs) and cluster 8 (containing tonsil DCs) (Supplementary Fig. 3A). Cluster 7 had higher expression for genes reported to be highly expressed in CD14^+^ monocytes, such as *TYROBP* (encoding DAP12)^22^, *TNFSF13B* (encoding BAFF)^23^, *NMT1* (a gene essential for monocyte development)^24^, or genes upregulated when monocytes differentiate into DCs such as *CST7*^25^ and *CD1E*^12^ (Supplementary Fig. 3B). By contrast, cluster 8 had higher expression of genes preferentially detected in other clusters of tonsil cDCs such as *RELB*, *FAM60A*, *IER2*, *TNFAIP2*, *SPI1*, *PTP4A2* (Supplementary Fig. 3C). These genes were found in an independent study to be expressed at similar levels in circulating cDCs from blood and resident cDCs from spleen (by both cDC1 and cDC2) (Supplementary Fig. 3D)^18^, indicating that their differential expression between clusters 7 and 8 is more likely related to distinct ontogeny rather than tissue type. This analysis suggests that cluster 7 corresponds to mo-DCs rather than cDCs.

Based on these results, we annotated cluster 1 as monocytes, clusters 2 and 3 as mo-Mac, cluster 4 as monocyte-derived cells at an early stage of differentiation, clusters 5 and 6 as mo-DCs, cluster 7 as end-stage mo-DCs, cluster 8 as activated cDC2, cluster 9 as cDC2, cluster 10 as contaminating tonsil macrophages, cluster 11 as cDC1, and clusters 12 and 13 as precursor cells (Fig. 2b).

Collectively, these results show that ascites CD1c^+^ DCs do not contain a population of cDCs and support their identification as in vivo-generated mo-DCs.

### Human mo-DCs and mo-Mac can both efficiently cross-present

**Fig. 3:**
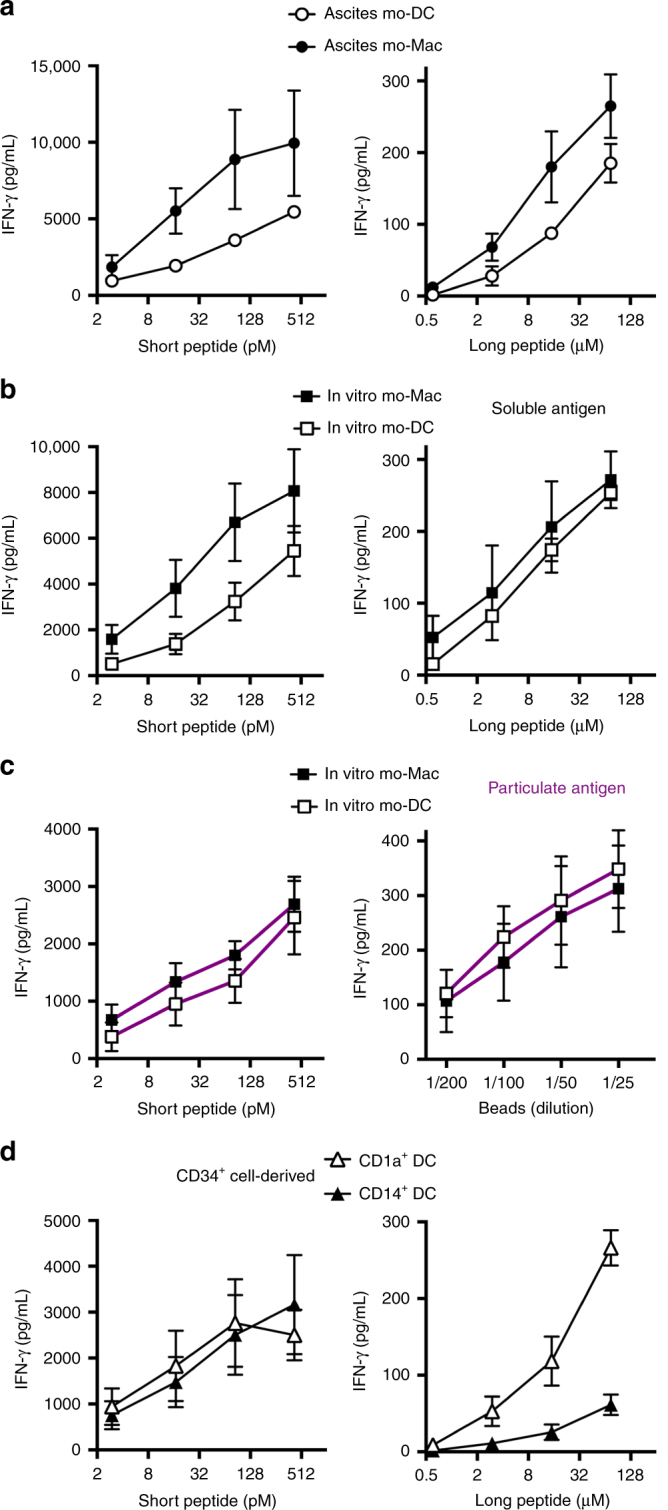
Human mo-DCs and mo-Mac both cross-present efficiently. Purified DCs and macrophages from tumor ascites (**a**) or in vitro culture of monocytes (**b**, **c**), or DCs derived in vitro from CD34^+^ precursors (**d**) were incubated with serial concentrations of MelanA long or short peptide (**a**, **b**, **d**) or MelanA-coated beads (**c**). After washing, antigen-specific CD8^+^ T cells were added. After 24 h, IFN-γ secretion was assessed as a measure of T cell activation. Background level was subtracted. Mean ± SEM of three (**a**, **d**), six (**b**), or five (**c**) independent experiments

To address whether ascites mo-DCs can cross-present, we analyzed cross-presentation of a model antigen using a MelanA-specific CD8^+^ T cell clone (HLA-A2-restricted). Ascites mo-DCs, and mo-Mac for comparison, were incubated with a 34-aa long peptide (requiring processing for cross-presentation) or a pre-processed short peptide corresponding to the minimal epitope, as control for T cell activation ability (Fig. 3a). Ascites mo-DCs and mo-Mac could both cross-present the MelanA antigen, with ascites mo-Mac being more efficient than mo-DCs. mo-Mac were also more efficient for presentation of the short peptide, suggesting a better ability for T cell activation, possibly due to greater MHC class I molecules expression. We also compared the relative expression of genes involved in antigen processing and presentation using Gene Set Enrichment Analysis. Consistent with results from the cross-presentation assay, we did not find any enrichment for gene signatures of antigen presentation between ascites mo-DCs and mo-Mac, although both cell types were enriched for these signatures compared to blood monocytes (Supplementary Fig. 4A–B).

This finding was surprising because we have previously shown, using the same model antigen, that tonsil macrophages are poor cross-presenting cells^9^. To assess whether the ability to cross-present was induced in macrophages by the ascites environment, we performed the same experiment using in vitro equivalents of these cells, obtained by culturing monocytes with M-CSF, IL-4, and TNFa^12^. In vitro-derived mo-DCs and mo-Mac could both cross-present MelanA antigen (Fig. 3b). mo-Mac were again more efficient for presentation of the short peptide, due to higher expression of the MHC class I molecule HLA-A2 (Supplementary Fig. 4C). We confirmed this result using MelanA-coated beads as a model for particulate antigen (Fig. 3c). As an internal control for this assay, we used DCs obtained by culturing blood CD34^+^ precursors with GM-CSF, Flt3-L, and TNFa. CD1a^+^ DCs could cross-present efficiently, in contrast to CD14^+^ DCs (Fig. 3d), as previously reported^9,26^. We conclude that mo-DCs and mo-Mac both have the intrinsic ability to cross-present antigens.

### mo-DCs and mo-Mac are poor for endosome-to-cytosol transfer

**Fig. 4:**
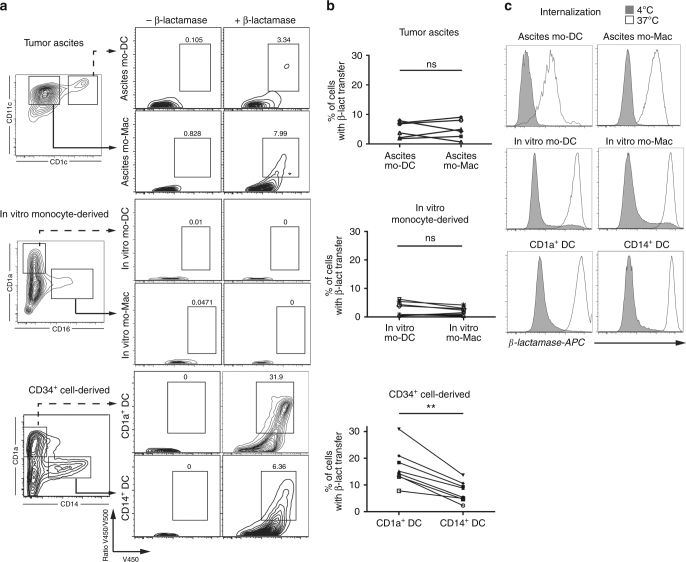
Human mo-DCs and mo-Mac are inefficient for the transfer of exogenous proteins into their cytosol. **a**, **b** Purified DCs and macrophages from tumor ascites, derived in vitro from monocytes, or DCs derived in vitro from CD34^+^ precursors were loaded with a cell-permeable FRET-sensitive substrate of β-lactamase, and incubated with or without exogenous β-lactamase. After 3 h, cleavage was measured by flow cytometry. **a** Representative results of six (tumor ascites), ten (in vitro monocyte-derived), or eight (in vitro CD34^+^ cell-derived) independent experiments. **b** Quantification of β-lactamase transfer. Symbols represent individual donors. *N* = 6 for tumor ascites, *N* = 10 for in vitro monocyte-derived cells, and *N* = 8 for CD34^+^ cell-derived cells. **p < 0.01, Wilcoxon non-parametric test. **c** Purified DCs or macrophages were incubated with β-lactamase coupled to Atto dye 633 at 4 or 37 °C during 3 h. Representative results of three independent experiments

Two main pathways have been described for cross-presentation^1,3^. In the “cytosolic pathway”, exogenous antigens are transferred from endocytic compartments into the cytosol, where they are degraded by the proteasome. In the “vacuolar pathway”, internalized antigens are degraded in endocytic compartments by lysosomal proteases. To address the intracellular pathway used by mo-DCs and mo-Mac for cross-presentation, we first analyzed their ability to transfer exogenous β-lactamase into their cytosol by measuring the cleavage of a cytosolic β-lactamase-sensitive FRET probe^9,27^. Ascites mo-DCs and mo-Mac were both poor at transferring exogenous β-lactamase into their cytosol (Fig. 4a, b). This was not due to the tumor ascites micro-environment, as the same was found for in vitro-differentiated mo-DCs and mo-Mac (Fig. 4a, b), nor to deficient uptake of β-lactamase, as all population could efficiently internalize fluorescent β-lactamase (Fig. 4c). By contrast, CD1a^+^ DCs could transfer exogenous β-lactamase into their cytosol more efficiently than CD14^+^ DCs, as previously reported^9^ (Fig. 4a, b). These results suggest that human mo-DCs and mo-Mac do not use the cytosolic pathway for cross-presentation.

### mo-DCs and mo-Mac use the vacuolar pathway

**Fig. 5:**
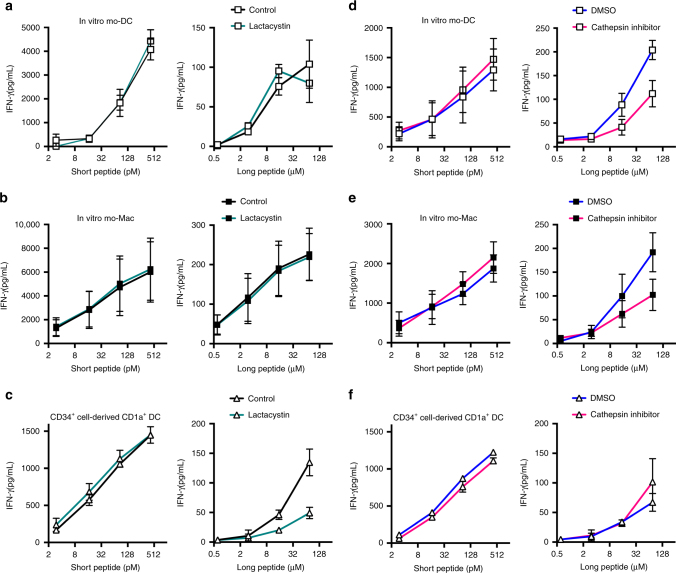
Human mo-DCs and mo-Mac use the vacuolar pathway for cross-presentation. Purified in vitro-generated mo-DCs (**a**, **d**), mo-Mac (**b**, **e**), or CD34^+^ cell-derived CD1a^+^ DCs (**c**, **f**) were incubated with serial concentrations of MelanA long or short peptide, in the absence or presence of lactacystin (**a**–**c**) or pan-cathepsin inhibitor (**d**–**f**). After washing, antigen-specific CD8^+^ T cells were added. After 24 h, IFN-γ secretion was assessed as a measure of T cell activation. Background level was subtracted. Mean ± SEM of three (**a**), five (**b**, **d**, **e**), or three (**c**, **f**) independent experiments

To confirm this finding, we analyzed cross-presentation by mo-DCs and mo-Mac in the presence of a proteasome inhibitor, lactacystin (Fig. 5a, b). Cross-presentation by mo-DCs or mo-Mac was not impaired in the presence of lactacystin. By contrast, lactacystin inhibited cross-presentation by CD1a^+^ DCs, as previously reported^9^ (Fig. 5c). To confirm that proteasome activity was inhibited by lactacystin in mo-DCs and mo-Mac at the concentration used in the cross-presentation assay, we performed a fluorometric assay for the chymotrypsin-like activity of the proteasome (Supplementary Fig. 5). Lactacystin significantly inhibited proteasome activity in both mo-DCs and mo-Mac. Collectively, these results show that proteasome activity was dispensable for cross-presentation by mo-DCs and mo-Mac.

To directly assess the role of the vacuolar pathway, we used a pan-cathepsin inhibitor to block the activity of lysosomal cysteine proteases. Transcriptomic analysis showed that mo-Mac express overall higher levels of lysosomal proteases than mo-DCs (Supplementary Fig. 6A). In the presence of the cathepsin inhibitor, cross-presentation by mo-DCs and mo-Mac was impaired compared to vehicle control (Fig. 5d, e). This was not due to toxicity of the inhibitor as cell viability was similar in all conditions (Supplementary Fig. 6B). In addition, cross-presentation by CD1a^+^ DCs was not affected by the cathepsin inhibitor (Fig. 5f). These results show that, in mo-DCs and mo-Mac, antigens are degraded by lysosomal proteases for cross-presentation.

We conclude that human monocyte-derived cells use exclusively the vacuolar pathway for cross-presentation.

### Only mo-DCs are efficient inducers of cytotoxic CD8^+^ T cells

**Fig. 6:**
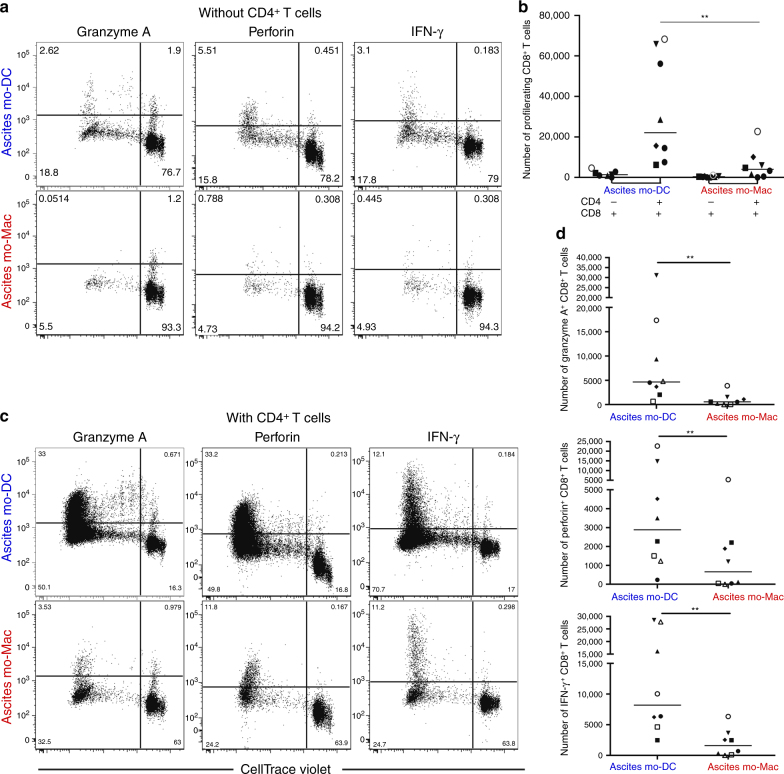
Human mo-DCs, but not mo-Mac, are efficient inducers of effector cytotoxic CD8^+^ T cells. Purified DCs and macrophage from tumor ascites were cultured with allogeneic CellTrace Violet-stained naïve CD8^+^ T cells for 7 days, in the absence (**a**, **b**) or presence (**c**, **d**) of naïve CD4^+^ T cells autologous to CD8^+^ T cells. Expression of Granzyme A, Perforin, and IFN-γ was assessed by intracellular flow cytometry. **a**, **c** Representative results of eight independent experiments. Gated on live CD8^+^ T cells. **b** Number of proliferating CD8^+^ T cells is shown. Symbols represent individual donors. *N* = 8. Median is shown. **d** Number of CD8^+^ T cells expressing effector molecules is shown. Symbols represent individual donors. *N* = 8. Median is shown. *p < 0.05, **p < 0.01, Wilcoxon non-parametric test

To address the outcome of cross-presentation, we analyzed the ability of ascites mo-DCs and mo-Mac to induce the differentiation of cytotoxic effectors from naïve CD8^+^ T cells. For this assay, we turned to an allogeneic culture system. We co-cultured purified mo-DCs or mo-Mac with allogeneic naïve CD8^+^ T cells, and assessed T cell proliferation and expression of effector molecules (Granzyme A, Perforin, and IFN-γ). Help from CD4^+^ T cells is necessary for the differentiation of effector cytotoxic CD8^+^ T cells^28–30^. In the setting we used, CD4^+^ T cells have been reported to be essential for CD8^+^ T cell proliferation^10^, which we confirmed (Fig. 6a, b). When cultured with both naïve CD4^+^ and CD8^+^ T cells, only mo-DCs could induce significant proliferation of CD8^+^ T cells and expression of Granzyme A, Perforin, and IFN-γ (Fig. 6c, d). To evaluate the efficiency of effector differentiation induced by mo-DCs, we performed the same experiment with tonsil DC subsets. We purified cDC1, cDC2, pDCs, and tonsil macrophages^9^. cDC1 and cDC2 were the most efficient inducers of CD8^+^ T cell proliferation and effector differentiation, while macrophages and pDCs were poor at it (Supplementary Fig. 7). Overall, proliferation and induction of effector molecules were comparable between cDC1, cDC2, and ascites mo-DCs, suggesting that mo-DCs are indeed efficient activators of cytotoxic CD8^+^ T cells.

**Fig. 7:**
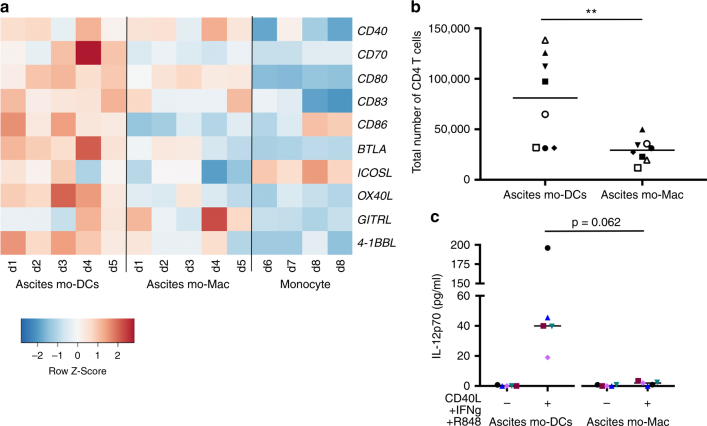
Human mo-DCs, but not mo-Mac, provide co-stimulatory signals for the differentiation of cytotoxic CD8^+^ T cells. **a** Heatmap of scaled expression for selected co-stimulation genes. d donor. **b** Purified DCs and macrophage from tumor ascites were cultured with allogeneic naïve CD4^+^ T cells for 7 days, in the presence of naïve CD8^+^ T cells. Total number of live CD4^+^ T cells at the end of the culture is depicted. *N* = 8. **c** Purified DCs and macrophage from tumor ascites were cultured in the absence or presence of CD40-L, IFN-γ, and R848 for 24 h. Secretion of IL-12p70 was measured in the supernatant. *N* = 5. **b**, **c** Symbols represent individual donors. Median is shown. ** *p* < 0.01, Wilcoxon non-parametric test

Finally, to address the mechanisms underlying the superior ability of ascites mo-DCs to prime effector CD8^+^ T cells, we compared the capacity of ascites mo-DCs and mo-Mac to provide co-stimulatory signals. Transcriptome analysis showed that ascites mo-DCs express higher levels of co-stimulatory molecules than ascites mo-Mac (Fig. 7a). Consistent with this, in our allogeneic culture system, mo-DCs were better stimulators of naïve CD4^+^ T cell proliferation (Fig. 7b), potentially inducing more CD4^+^ T cell help. To address the ability of ascites mo-DCs and mo-Mac to secrete cytokines involved in the acquisition of CD8^+^ T cell effector functions^31^, we measured the production of IL-12p70 after ex vivo restimulation. Only ascites mo-DCs were able to secrete IL-12p70 (Fig. 7c). Collectively, these results indicate that only mo-DCs, but not mo-Mac, are able to provide the co-stimulatory signals necessary for efficient cytotoxic CD8^+^ T cell differentiation.

## Discussion

Here, we show that human mo-DCs and mo-Mac, both naturally occurring in vivo in peritoneal ascites and generated in vitro from monocytes cultured with M-CSF, IL-4, and TNFα, cross-present exclusively using a vacuolar pathway. However, only ascites mo-DCs induce the differentiation of cytotoxic CD8^+^ T cells.

Numerous studies have shown that DCs are the most efficient cross-presenting cells^1,3^. However, mouse macrophages can cross-present in vitro^32–34^, and in vivo in some settings^35,36^. Human in vitro-generated macrophages can also cross-present antigens^37,38^, in contrast to macrophages isolated from tonsils^9^. Here, we found that human macrophages from tumor ascites can cross-present as efficiently as mo-DCs from the same samples. These discrepancies could be explained by functional differences related to the adaptation of macrophages to their tissue environment^39,40^.

Contradictory data exists on the pathway used for cross-presentation by in vitro-differentiated DCs derived from monocytes using GM-CSF and IL-4, with some studies showing for the cross-presentation of soluble antigens a vacuolar pathway^41,42^ and others a cytosolic pathway^38,43–45^, while cross-presentation of cell-associated antigen was reported to be proteasome-dependent^46^. The reasons for these discrepancies are not clear. Using a culture model that yields mo-DCs closely resembling in vivo-generated mo-DCs^12^, we found that mo-DCs were inefficient for transferring exogenous proteins into their cytosol, and use a vacuolar pathway for cross-presentation.

It has been proposed that the choice of cytosolic versus vacuolar pathway for cross-presentation is dictated by the form of antigen. Consistent with this, human pDCs cross-present soluble and cell-associated antigens using a cytosolic pathway^9,47^, but cross-presentation of viral antigens has been shown to be proteasome-independent and to use a vacuolar pathway^48^. Furthermore, human cDC1 cross-present soluble antigens using a cytosolic pathway^9,42,49^, but cross-presentation of immune complexes is inhibited by both proteasome and lysosomal proteases inhibitors^50^. The possibility to use either pathway for cross-presentation may also be subset-specific. In support of this, the molecular requirements for cross-presentation are different between mouse cDC1 and mo-DCs both in vivo and in vitro^51,52^. Our results extend these observations to human DC subsets.

Mouse cDC1 are recognized as the main cross-presenting cells^1,3^. mo-DCs can also cross-present efficiently in various inflammatory settings^4–7,51,53,54^. Accumulating evidence indicate that mo-DCs play a key role in the induction and regulation of cytotoxic T cell responses, complementary to that of cDC1. In contrast to cDC1 that interact with CD8^+^ T cells in lymphoid organs, mo-DCs are able to cross-present antigens and to stimulate effector CD8^+^ T cells directly in situ, in inflammed tissues^4–6^. In addition, mouse mo-DCs express high levels of co-stimulatory signals involved in the differentiation of cytotoxic CD8^+^ T cells, including CD70^54,55^. Our results suggest that human in vivo-generated mo-DCs are equipped for playing a similar role.

Enhancing cross-presentation represents a way of improving vaccination efficiency or anti-tumor immune responses. Deciphering cross-presentation in human DCs that are present in vivo is a pre-requisite for its manipulating for therapeutic purposes. By providing a better understanding of cross-presentation mechanisms in human DC subsets, our results should have important implications for the design of DC-targeted therapies.

## Methods

### Human samples

Buffy coats from healthy donors (both male and female donors) were obtained from Etablissement Français du Sang (Paris, France) in accordance with INSERM ethical guidelines. Tumor ascites from ovarian cancer patients were obtained from Hôpital de l’Institut Curie in accordance with hospital guidelines. Tonsils from healthy patients (both male and female) undergoing tonsillectomy were obtained from Hôpital Necker (Paris, France). According to French Public Health Law (art L 1121-1-1, art L 1121-1-2), written consent and IRB approval are not required for human non-interventional studies.

### Cell isolation

Tonsil samples were digested as described previously^56^. In brief, samples were cut into small fragments, digested with 0.1 mg mL^−1^ Liberase TL (Roche) in the presence of 0.1 mg mL^−1^ DNAse (Roche) for 40 min at room temperature before addition of 10 mM EDTA. Cells were filtered on a 40 μm cell strainer (BD Falcon) and washed. Light density cells were isolated by centrifugation on a Ficoll gradient (Lymphoprep, Greiner Bio-One). DCs were enriched by depletion of cells expressing CD3, CD15, CD19, CD56, and CD235a using antibody-coated magnetic beads (Miltenyi). Cell subsets were further isolated by cell sorting on a FACSAria instrument after staining for CD11c, HLA-DR, CD14, CD304, CD1c, and CD141 (BD Biosciences). Peripheral blood mononuclear cells (PBMC) were prepared by centrifugation on a Ficoll gradient. Blood CD14^+^ monocytes were isolated from healthy donors’ PBMC by positive selection using anti-CD14-coated magnetic beads according to manufacturer’s instructions (Miltenyi). DCs and macrophage population from ascites were isolated after centrifugation of total ascites cells on a Ficoll gradient, enrichment by depletion of cells expressing CD3, CD15, CD19, CD56, and CD235a using antibody-coated magnetic beads (Miltenyi), and cell sorting on a FACSAria instrument. Ascites DCs were gated as HLA-DR^+^CD11c^+^CD1c^+^CD16^−^ and ascites macrophages as HLA-DR^+^CD11c^+^CD1c^−^CD16^+^.

### Cell culture

Blood CD34^+^ cells were isolated from PBMC by positive selection using anti-CD34-coated magnetic beads and magnetic columns according to manufacturer’s instructions (Miltenyi). CD34^+^ cells were cultured for 9–10 days in Yssel medium supplemented with 10% fetal calf serum (FCS), penicillin/streptomycin, 50 ng mL^−1^ GM-CSF (Miltenyi), 100 ng mL^−1^ Flt3-L (Miltenyi), and 10 ng mL^−1^ TNF-α (Miltenyi). DC subsets were isolated by cell sorting on a FACSAria instrument (BD Biosciences) after staining for CD1a and CD14. Monocytes (1×10^6^ cells mL^−1^) were cultured for 5 days in RPMI-Glutamax medium (Gibco) supplemented with antibiotics (penicillin and streptomycin) and 10% FCS in the presence or absence of 100 ng mL^−1^ M-CSF (Miltenyi), 40 ng mL^−1^ IL-4 (Miltenyi), and 5 ng mL^−1^ TNF-α (Miltenyi). Cell population was isolated by cell sorting on a FACSAria instrument after staining for CD1a and CD16.

### Flow cytometry

Non-specific binding was blocked using TruStain (Biolegend). Unless otherwise stated, cell viability was assessed using DAPI (Sigma). Cells were stained with FITC anti-CD16 (catalog #555406, BD Bioscience, dilution 1/100), APC anti-CD16 (catalog #302012, Biolegend, dilution 1/200), APC anti-CD1a (catalog #300110, BioLegend, dilution 1/200), APC-Vio770 anti-CD1a (catalog #130-105-527, Miltenyi, dilution 1/100), FITC anti-CD14 (catalog #11-0149-42, eBioscience, dilution 1/100), APC-Vio770 anti-CD14 (catalog #130-098-076, Miltenyi, dilution 1/100), PerCP/Cy5.5 anti-CD1c (catalog #331514, BioLegend, dilution 1/100), APC anti-CD1c (catalog #331524, BioLegend, dilution 1/100), APC-eFluor780 anti-HLA-DR (catalog #47-9956-42, eBioscience, dilution 1/100), Pe/Cy7 anti-CD11c (catalog #337216, BioLegend, dilution 1/100), APC anti-CD123 (catalog #130-090-901, Miltenyi Biotec, dilution 1/100), PE anti-BDCA3/CD141 (catalog #130-090-514, Miltenyi Biotec, dilution 1/100), PE anti-HLA-A2 (catalog #558570, BD, dilution 1/25), FITC anti-TCR Vbeta2 (catalog #IM2407, Beckman coulter, dilution 1/50), Alexa Fluor 488 anti-Granzyme A (catalog #507212, BioLegend, dilution 1/100), PE/Cy7 anti-IFN-γ (catalog #25-7319-82, eBioscience, dilution 1/50), PerCP/Cy5.5 anti-CD8 (catalog #344710, BioLegend, dilution 1/200), APC anti-CD4 (catalog #300514, BioLegend, dilution 1/200), and PE anti-Perforin (catalog #308106, BioLegend, dilution 1/100). Cells were analyzed on a FACSVerse instrument (BD Biosciences).

### Cytosolic translocation assay

Cells (10 × 10^6^ cells/condition for ascites cells and 5 × 10^6^ cells/condition for in vitro-generated cultures) were incubated with 0.5 μg mL^−1^ CCF4-AM (Invitrogen) for 30 min at room temperature at 10 × 10^6^ cells mL^−1^ in loading buffer (120 mM NaCl, 7 mM KCl, 1.8 mM CaCl_2_, 0.8 mM MgCl_2_, 5 mM glucose, 25 mM Hepes, pH 7.3) containing solution B (dilution 1/20, LiveBLAzer FRET-B/G loading kit, Invitrogen) and 1 mM probenecid (Invitrogen). After washing, cells were incubated at 10 × 10^6^ cells mL^−1^ in loading buffer containing 1 mM probenecid in the presence or absence of 2 mg mL^−1^ of β-lactamase (Penicillinase from *Bacillus cereus*, Sigma) for 3 h at 4 or 37 °C. Cell viability was assessed using Fixable Viability Dye eFluor780 (eBioscience). After washing, cells were stained for surface markers (CD11c-PeCy7 and CD1c-APC for ascites cells; CD1a-APC-Vio770 and CD16-APC for in vitro-generated monocyte-derived cells; CD1a-APC and CD14-APC-Vio770 for CD34^+^cells-derived cultures). Cells were analyzed on a FACSVerse Instrument (BD Biosciences). CCF4-AM fluorescence was assessed by measuring the 450 and 520 nm channels.

### Internalization assay

β-Lactamase was conjugated to Atto dye 633 according to manufacturer’s instructions (Sigma). Cells were incubated with fluorescent β-lactamase in Yssel medium at 4 or 37 °C for 3 h. After extensive washing, cells were analyzed with a FACSVerse Instrument.

### Cross-presentation assay

Antigen sources were MelanA short peptide (EAAGIGILTV), MelanA long peptide (KGHGHSYTTAEEAAGIGILTVILGVL), or beads coated with 750 μM of MelanA long peptide. In brief, Polybead 3-micron polystyrene microspheres (Polypeptide) were washed 3 times in PBS, then incubated at 4 °C overnight in PBS containing 750 μM of MelanA long peptide (in a volume 4 times that of the initial volume of the beads). Beads were washed 3 times in PBS and resuspended in PBS to their initial volume. Cross-presentation assay was performed as described^57^. In brief, purified HLA-A2^+^ APCs were incubated (10^4^ cells per well) for 3–4 h in Yssel medium in V-bottom 96-well plates (Corning) with different concentrations of MelanA long peptide, MelanA short peptide, or MelanA-coated beads, in the presence or absence of 2.5 μg mL^−1^ lactacystin (clasto-Lactacystin β-Lactone; Merck/Millipore), 10 μM Cathepsin Inhibitor I (inhibiting cathepsin B, cathepsin L, cathepsin S, and papain; Calbiochem) or the corresponding concentration of DMSO (Sigma). After extensive washing, APCs were cultured for 24 h with CD8 T cell LT12 clones^58^ (2×10^4^ cells per well) in Yssel medium supplemented with 10% FCS. Supernatants were collected and kept at −20 °C until measurement of IFN-γ concentration by ELISA (BD Biosciences). Background levels (APC cultured with LT12 cells without peptide) was substracted for each cell type.

### Proteasome activity assay

Proteasome activity was assessed using a proteasome 20S activity kit (Sigma). For proteasome activity assay, mo-DCs and mo-Mac from in vitro cultures were purified using anti-CD16- or anti-CD1a-coated magnetic beads and magnetic columns according to manufacturer’s instructions (Miltenyi). Cells (10^5^ cells per well, triplicate wells) were incubated with or without 2.5 μg mL^−1^ lactacystin for 30 min at 37 °C in RPMI supplemented with 10% FCS. Cells were then incubated with the assay loading solution for 3 h at 37 °C. Fluorescence ratio between 490 nm (excitation) and 525 nm (emission) was measured using a FLUOstar Omega instrument (BMG Labtech). Background fluorescence of blank wells (medium without cells) was substracted.

### Cytotoxic T lymphocyte polarization assay

Naive CD8^+^ T cells and CD4^+^ T cells were isolated from healthy donors’ PBMC using EasySep human Naïve CD8 or CD4 isolation kit according to manufacturer’s instructions (StemCell Technologies). APC (1×10^4^ cells per well) were cultured with naive CD8^+^ T cells (5×10^4^ cells per well) with or without naive CD4^+^ T cells from the same T cell donor (5×10^4^ cells per well) for 7 days in Yssel medium supplemented with 10% FCS. To analyze T cell proliferation, CD8^+^ T cells were stained with Cell Trace Violet (CTV, Thermo Fisher) prior to culture. To assess the expression of intracellular effector molecules, T cells were stimulated with PMA (50 ng mL^−1^) and ionomycin (1 µg mL^−1^) for 6 h in the presence of BFA (4 µg mL^−1^) for 6 h (all from Sigma). After washing, cells were stained for surface CD4 for 30 min at 4 °C, washed and stained with Live/dead eFluor780 (Thermo Fisher Scientific) for 20 min at 4 °C. Then the cells were fixed and permeabilized (Intracellular Fixation & Permeabilization Buffer Set, eBioscience) and stained for intracellular proteins (Granzyme A, Perforin, and IFN-γ) at room temperature for 45 min in a buffer containing 2% of normal mouse serum. The samples were acquired on a FACSVerse instrument (BD Biosciences).

### Cytokine secretion

Sorted cell population (2.5×10^4^ cells per well) were incubated during 24 h in Yssel medium in the absence or presence of 1 μg mL^−1^ dimerized CD40-ligand (Alexis), 1000 IU mL^−1^ IFNγ (Miltenyi), and 1 µg mL^−1^ R848 (Invivogen). Supernatants were collected and kept at −20 °C. IL12p70 secretion was assessed by CBA (BD Biosciences).

### Gene expression analysis

For differential gene expression analysis of ascites mo-DCs and mo-Mac and blood monocytes, we used transcriptomic data from Affymetrix microarrays that we had previously generated (GSE40484)^13^. Analysis was performed using R (v3.3.3). Raw data was preprocessed using the Robust Multi-array Average (RMA) method from *oligo* package^59^. Gene expression levels were analyzed on a base-2 logarithmic scale. Moderated *t*-tests were performed using the *limma* package^60^ and the *p*-values were corrected for multiple testing with the Benjamini Hochberg method. Heatmaps were produced using R package *gplots*. For gene expression analysis of blood and spleen cDC1 and cDC2, we used published datasets (GSE77671)^18^.

### Gene Set Enrichment Analysis

GSEA^61^ was performed using the GSEA software (v6.3)^62^ and gene signatures from MSigDB (v6.1)^63^. GSEA has been performed with the default parameters except for the number of permutations that we fixed at *n* = 1000 and the number of min gene at *n* = 15.

### Single-cell RNA-seq library preparation

Cellular suspension (3500 cells) of cell-sorted ascites DCs (gated as HLA-DR^+^CD11c^+^CD1c^+^CD16^−^), ascites macrophages (gated as HLA-DR^+^CD11c^+^CD1c^−^CD16^+^), or tonsil DCs (gated as HLA-DR^+^CD11c^+^CD14^−^) was loaded on a 10× Chromium instrument (10× Genomics) according to manufacturer’s protocol based on the 10× GEMCode proprietary technology. Single-cell RNA-Seq libraries were prepared using Chromium Single Cell 3′ v2 Reagent Kit (10× Genomics) according to manufacturer’s protocol. Briefly, the initial step consisted in performing an emulsion where individual cells were isolated into droplets together with gel beads coated with unique primers bearing 10× cell barcodes, unique molecular identifiers (UMI), and poly(dT) sequences. Reverse transcription reactions were engaged to generate barcoded full-length cDNA followed by the disruption of emulsions using the recovery agent and cDNA clean up with DynaBeads MyOne Silane Beads (Thermo Fisher Scientific). Bulk cDNA was amplified using a GeneAmp PCR System 9700 with 96-Well Gold Sample Block Module (Applied Biosystems) (98 °C for 3 min; cycled 14×: 98 °C for 15 s, 67 °C for 20 s, and 72 °C for 1 min; held at 4 °C). Amplified cDNA product was cleaned up with the SPRI select Reagent Kit (Beckman Coulter). Indexed sequencing libraries were constructed using the reagents from the Chromium Single Cell 3′ v2 Reagent Kit, following these steps: (1) fragmentation, end repair, and A-tailing; (2) size selection with SPRI select; (3) adaptor ligation; (4) post ligation cleanup with SPRI select; (5) sample index PCR and cleanup with SPRI select beads. Library quantification and quality assessment was performed using Qubit fluorometric assay (Invitrogen) with dsDNA HS (High Sensitivity) Assay Kit and Bioanalyzer Agilent 2100 using a High Sensitivity DNA chip (Agilent Genomics). Indexed libraries were equimolarly pooled and sequenced on an Illumina HiSeq2500 using paired-end 26 × 98 bp as sequencing mode. Using a full Rapid flow cell, a coverage around 100 million reads per sample were obtained corresponding to 100,000 reads per cell.

### Single-cell RNA-seq data analysis

Single-cell expression was analyzed using the Cell Ranger Single Cell Software Suite (v2.0.1) to perform quality control, sample de-multiplexing, barcode processing, and single-cell 3′ gene counting^14^. Sequencing reads were aligned to the UCSC hg38 transcriptome using the Cell Ranger suite with default parameters. Samples were merged using Cellranger aggregate function with default parameters. A total of 8404 single cells were analyzed. Mean raw reads per cell were 59,333. Further analysis was performed in R (v3.4) using the Seurat package (v2.2.1)^15^. The gene-cell-barcode matrix of the samples was log-transformed and filtered based on the number of genes detected per cell (any cell with less than 400 genes or more than 5000 genes per cell was filtered out). Any cell with more than 6% of mitochondrial UMI counts and more than 50% of ribosomal UMI was filtered out. Regression in gene expression was performed based on the number of UMI and the percentage of mitochondrial genes. Only genes detected in at least three cells were included. Cells were then scaled to a total of 1^e4^ molecules. Altogether, 6964 cells were kept for statistical analysis. To reduce data dimensionality, 5789 variable genes were selected based on their expression and dispersion (expression cut-off = 0, and dispersion cut-off = 0.5). PCA was run on the normalized gene-barcode matrix. Barnes-hut approximation to *t*-SNE^64^ was then performed on the first 19 principal components to visualize cells in a two-dimensional space. The first 19 principal components were used for the *t*-SNE projection and clustering analysis using the Elbow Plot approach. Clusters were identified using the “Find_Clusters” function in Seurat with a resolution parameter of 0.8. This graph-based clustering method relies on a clustering algorithm based on shared nearest neighbor (SNN) modularity optimization. Unique cluster-specific genes were identified by running the Seurat “Find_All_Markers” function using the MAST framework^65^. Three clusters containing contaminating cells were removed from the analysis: a cluster of 65 cells from the tonsil DCs sample corresponding to NK T cells (top genes: *CTSW, KLRB1, CD7, TRDC, XCL2, XCL1, AC092580.4, GNLY, IL2RB, TRBC1, KLRC1, CD3E*), a cluster of 58 cells from the ascites DCs sample corresponding to inflammatory CD11c^+^ B lymphocytes (top genes: *IGKC, CD79A, JCHAIN, IGLC2, CPNE5, ISG20, CD79B, MZB1, MS4A1, IGHA1, IGHG3, AL928768.3*), a cluster of 13 cells from both tonsil DCs and ascites DCs samples corresponding to epithelial cells (top genes: *CCDC80, KRT18, TM4SF1, KRT8, CALD1, SLPI, PRG4, NNMT, PLA2G2A, KRT19, DSRN, C3*). Heatmaps and violin plots were plotted using Seurat. Data is available at GEO (accession numbers GSE115007 and GSE115006). Scripts used to perform this analysis are available on GitHub (https://github.com/p-gueguen/tang_et_al_2018).

### Analysis of gene signatures at the single-cell level

Signature scores were computed using the Seurat function “AddModuleScore” using the gene signature of interest. This function calculates for each individual cell the average expression of each gene signature, subtracted by the aggregated expression of control gene sets^16^. All analyzed genes are binned into 25 bins based on averaged expression, and for each gene of the gene signature, 100 control genes are randomly selected from the same bin as the gene. Featureplots were plotted using minimum and maximum cutoff values for each feature were respectively quantile 3 and quantile 97. We used published gene signatures for skin CD14^+^ cells, blood cDC1, blood cDC2, and blood CD14^+^ monocytes^17^. To design genes signature, we used the GeneSign module of BubbleGUM software^66^ with our transcriptomic data (GSE40484 and GSE102046)^13,12^. To extract genes enriched in blood cDC2 compared to ascites DCs and ascites macrophages, we used the Mean(Test)/Mean(Ref) method and cut-off of 1% for the adjusted *p*-value and 1.5 for fold change. To extract genes enriched in in vitro mo-DCs compared to in vitro mo-Mac and monocytes or in vitro mo-Mac compared to in vitro mo-DCs and monocytes, we used the Minimal Pairwise (Mean(Test)/Mean(Ref)) method and cut-off of 1% for the adjusted *p*-value and 2 for fold change. To design a gene signature for tissue cDC2, we used the GeneSign module of BubbleGUM software^66^ with published transcriptomic data (GSE77671)^18^, using the Minimal Pairwise (Mean(Test)/Mean(Ref)) method and cut-off of 1% for the adjusted *p*-value and 2 for fold change. To design a signature of genes upregulated upon DC activation, we use published gene expression data of blood cDC2 and in vitro-generated mo-DCs cultured with GM-CSF and IL-4, exposed to the same stimulus (GSE56744 and GSE44721)^19^. We identified the genes that are concomitantly (i) up-regulated genes in mo-DCs activated with Menomune (*Neisseria meningitidis* vaccine, MGL) compared to unstimulated, and (ii) up-regulated genes in cDC2 activated with MGL compared to unstimulated. Gene expression levels were analyzed on a base-2 logarithmic scale. Moderated *t*-tests were performed using the *limma* package^60^ and the *p*-values were corrected for multiple testing with the Benjamini Hochberg method. We used a cut-off of 1% for the adjusted *p*-value and 2 for fold change.

### Software and statistical analysis

Flow cytometry data were analyzed using FlowJo software v9.9 or v10 (Tree Star). Statistical analyses were performed using the Prism software v7 (GraphPad). Wilcoxon non-parametric test was used. Variance was similar between the groups being compared.

### Data availability

Single-cell RNA-seq data that support the findings of this study have been deposited in GEO with accession codes: GSE115007 for ascites DCs and ascites macrophages and GSE115006 for tonsil DCs.

## Electronic supplementary material

Supplementary Information

Peer Review Report

Description of Additional Supplementary Files

Supplementary Data 1
