# Supplementary material for: Human in vivo-generated monocyte-derived dendritic cells and macrophages cross-present antigens through a vacuolar pathway
Source: Nat Commun. 2018 Jul 2;9:2570. doi: 10.1038/s41467-018-04985-0 (PMC6028641; doi:10.1038/s41467-018-04985-0)
Supplement: Supplementary file 1 — Supplementary Information [file 41467_2018_4985_MOESM1_ESM.pdf]

Supplementary information

Human *in vivo*-generated monocyte-derived dendritic cells and macophages cross-present through a vacuolar pathway

Tang et al

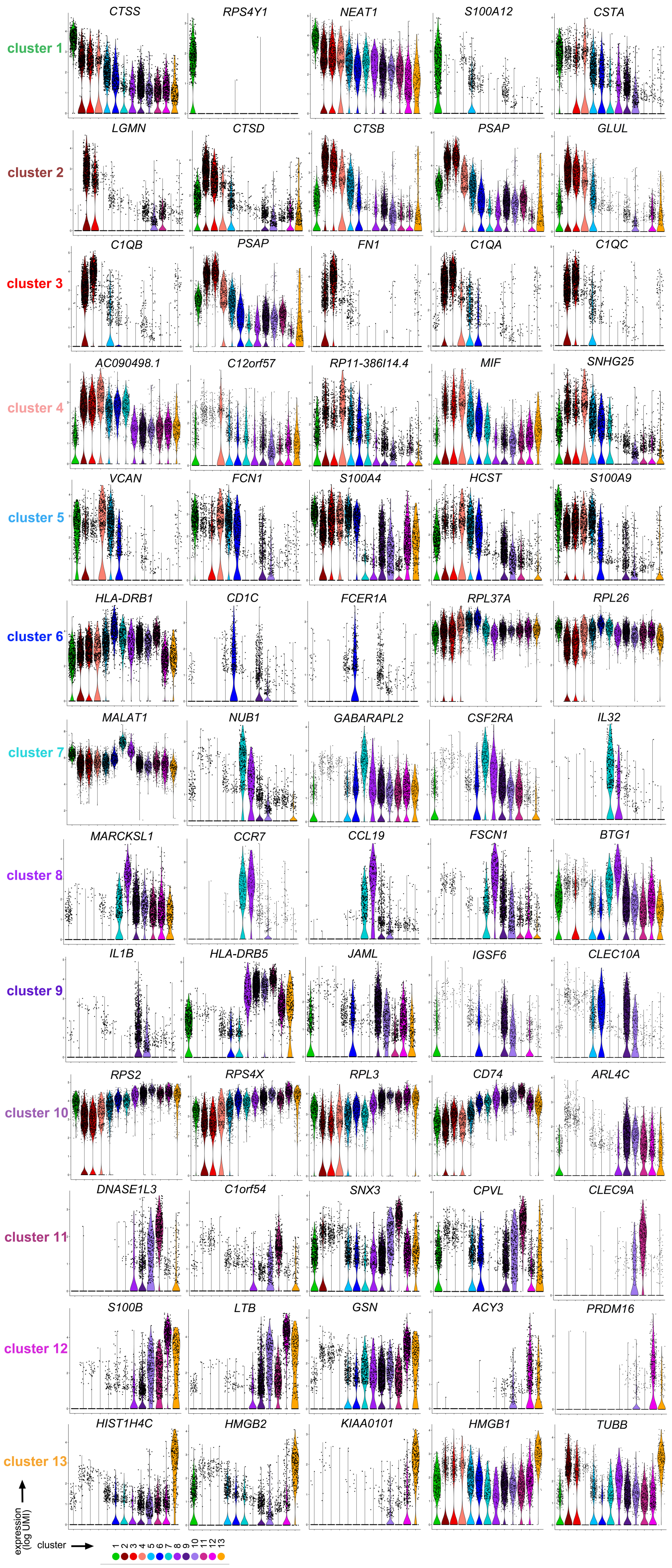

Supplementary Figure 1. Expression of the top differentially expressed genes for each cluster. Expression values (log values of UMI) for the top 5 most differentially expressed genes for each cluster (based on log fold change). Expression of these genes is depicted across all clusters.

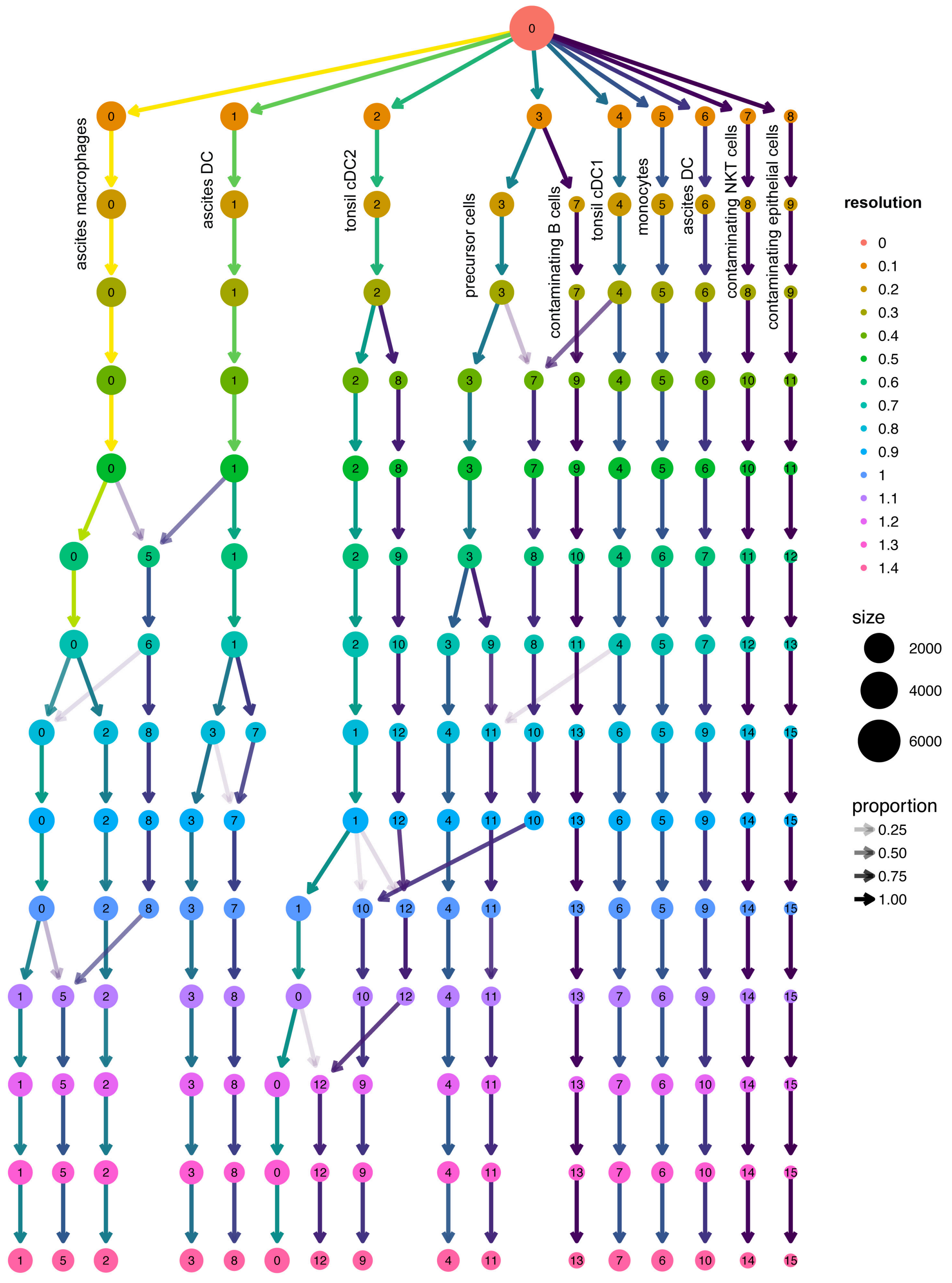

Supplementary Figure 2. Ascites DC and tonsil DC are not grouped in the same clusters independently of the resolution used. Clustree analysis [1] of the complete single-cell RNA-seq dataset, using results from Seurat with resolution parameters from 0 to 1.4. Clusters are numbered based on the number of cells they contain. The 10 main branches that form starting at resolution 0.2 have been annotated for cell identity.

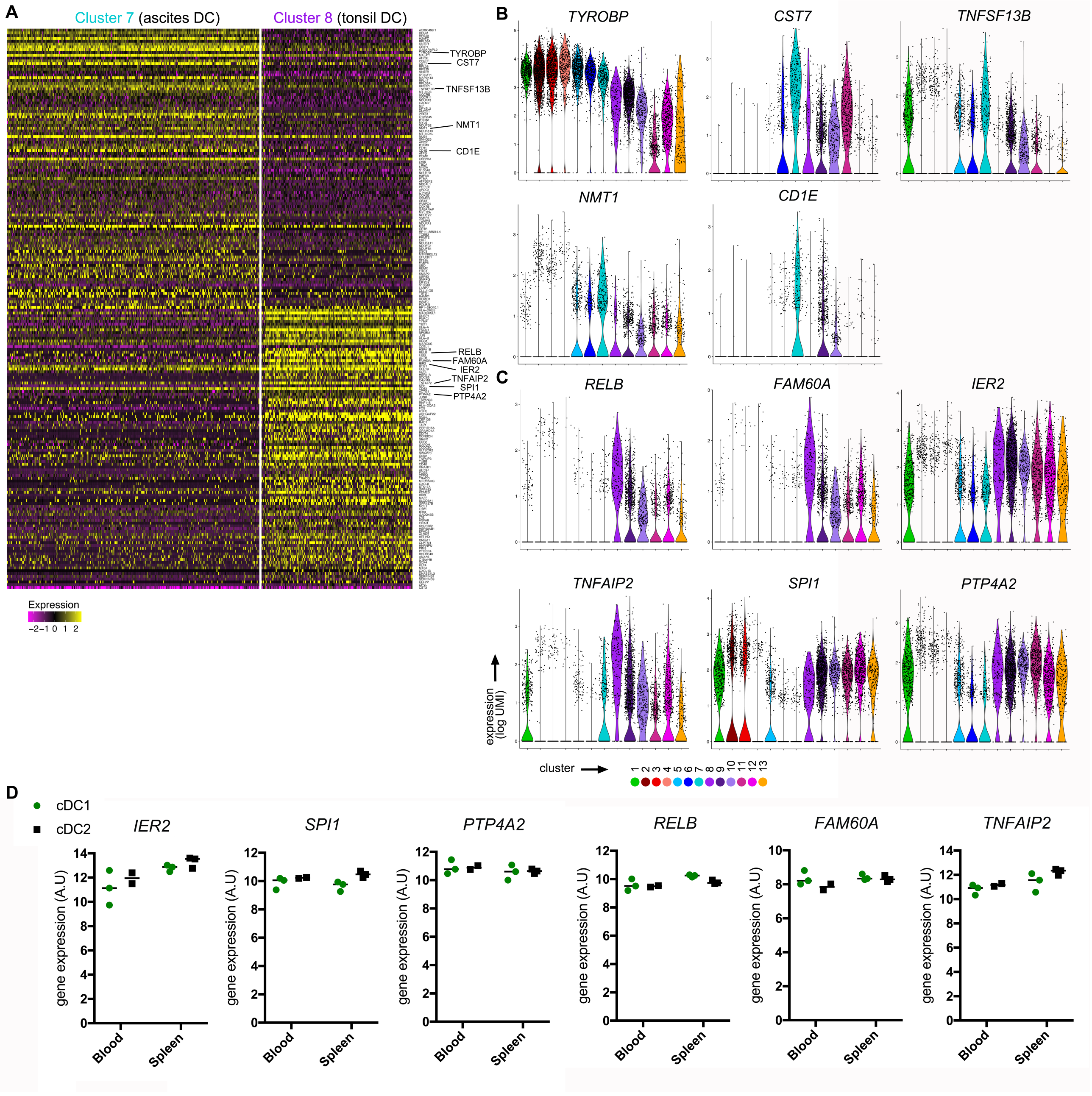

Supplementary Figure 3. Ascites DC are monocyte-derived cells. (A) Heatmap of scaled expression (log values of UMI) for the top 50 differentially expressed genes between clusters 7 and 8. (B-C) Scaled expression (log values of UMI) in individual cells for selected genes. (D) Expression of selected genes in human blood and spleen cDC. Expression values were extracted from public data (GSE77671). N=2 or 3. Median is shown.

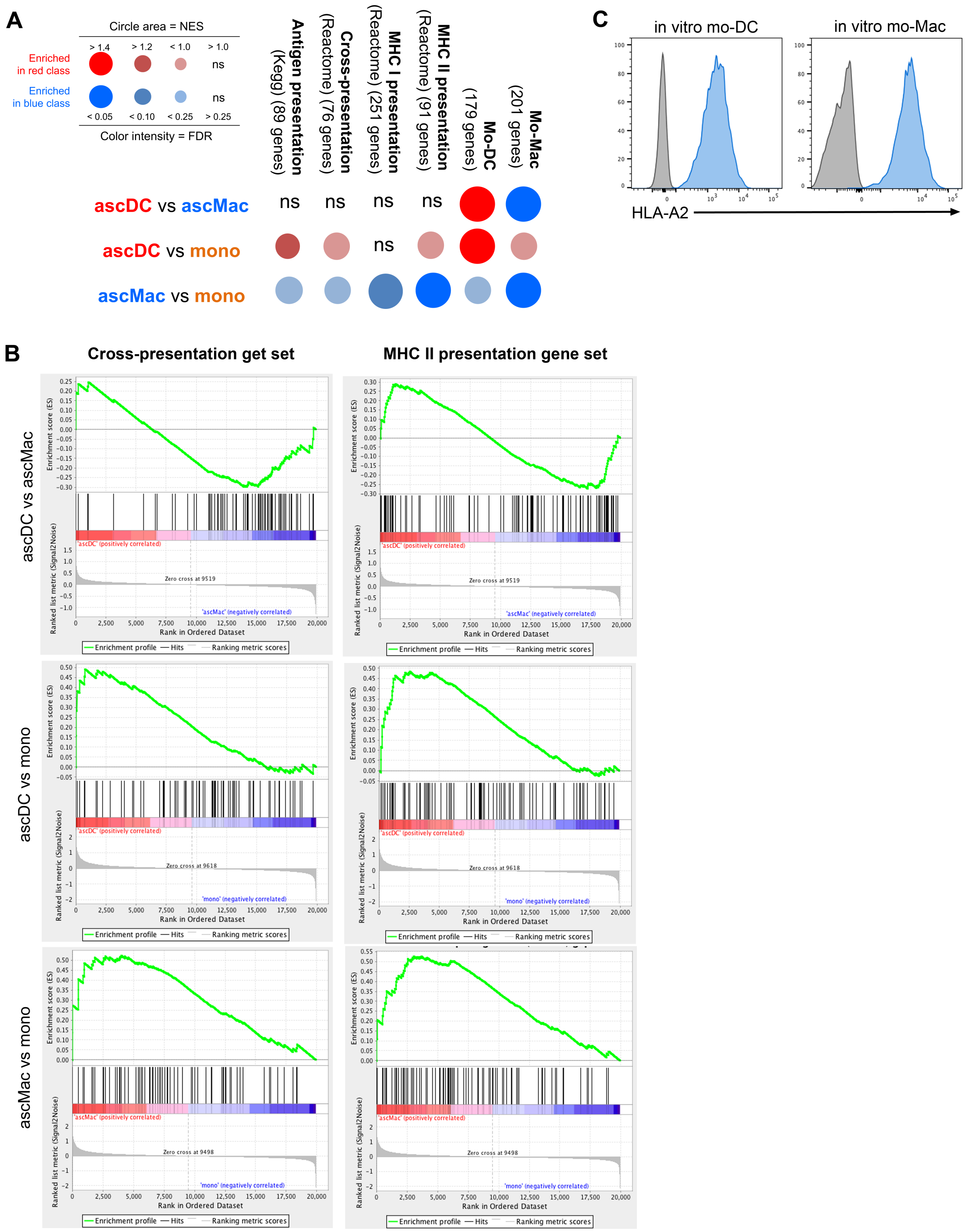

Supplementary Figure 4. Expression of antigen presentation genes by mo-DC and mo-Mac. (A-B) Gene set enrichment analysis (GSEA) analysis was performed on comparisons of ascites DC, ascites macrophages and blood monocytes for selected gene signatures. (A) Schematic BubbleGUM map of GSEA analysis is depicted. The color of bubbles indicates the population in which the signature is enriched. Strength of enrichment is represented by normalized enrichment score (NES). Significance of enrichment is represented by the false discovery rate (FDR). ns=not significant. (B) Gene set enrichment plots for the cross-presentation and MHC II presentation gene signatures. (C) *In vitro* mo-DC and *in vitro* mo-Mac were stained for HLA-A2 (blue histogram) or isotype control (gray histogram). Representative of 12 different donors.

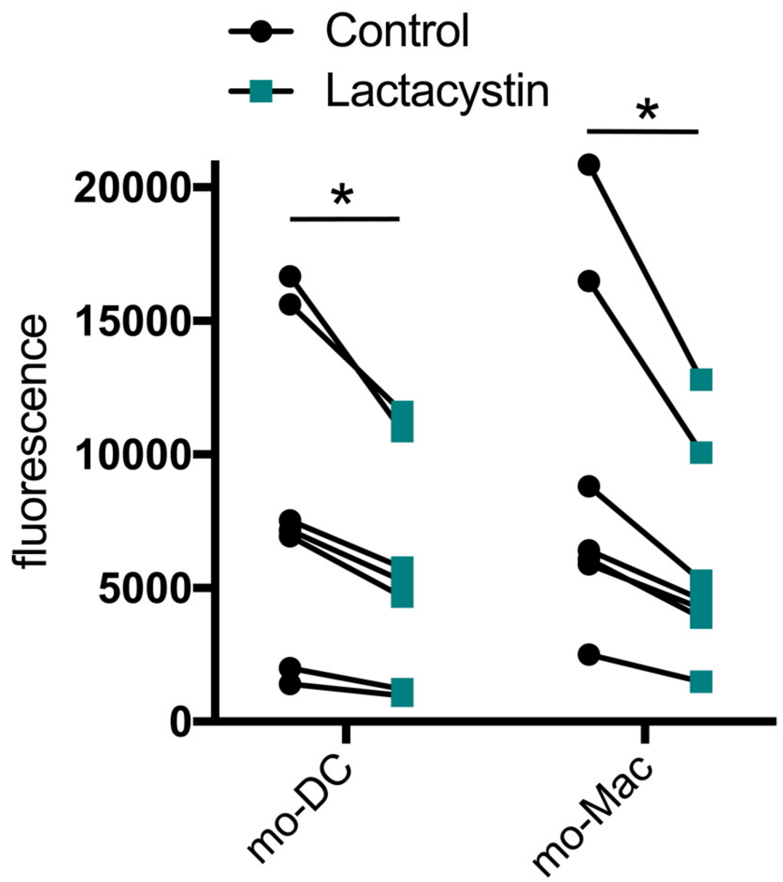

Supplementary Figure 5. Lactacystin inhibits the proteasome activity in mo-DC and mo-Mac. *In vitro* mo-DC and mo-Mac were incubated for 30 min in presence or absence of lactacystin. Proteasome activity was measured using a fluorometric assay. Fluorescence intensity after 3h is shown. N=7, * $p<0.05$, Wilcoxon non-parametric test.

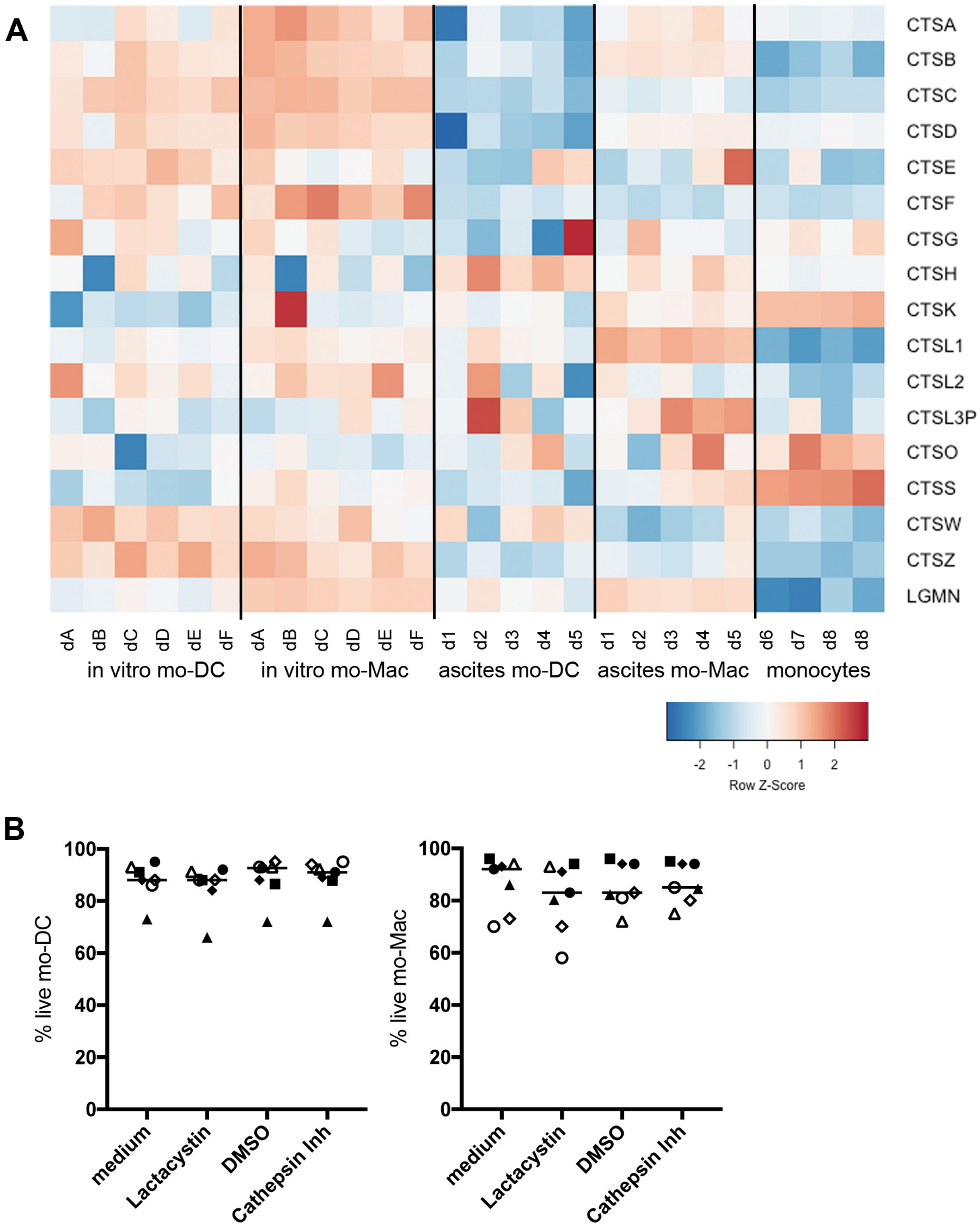

Supplementary Figure 6. Expression of lysosomal proteases. (A) Heatmap of scaled expression for lysosomal proteases genes. d=donor. (B) *In vitro* mo-DC and mo-Mac were cultured in the presence or absence of Lactacystin, DMSO or Cathepsin Inhibitor. Percentage of live cells after 3h is depicted. Symbols represent individual donors. N=7. Median is shown.

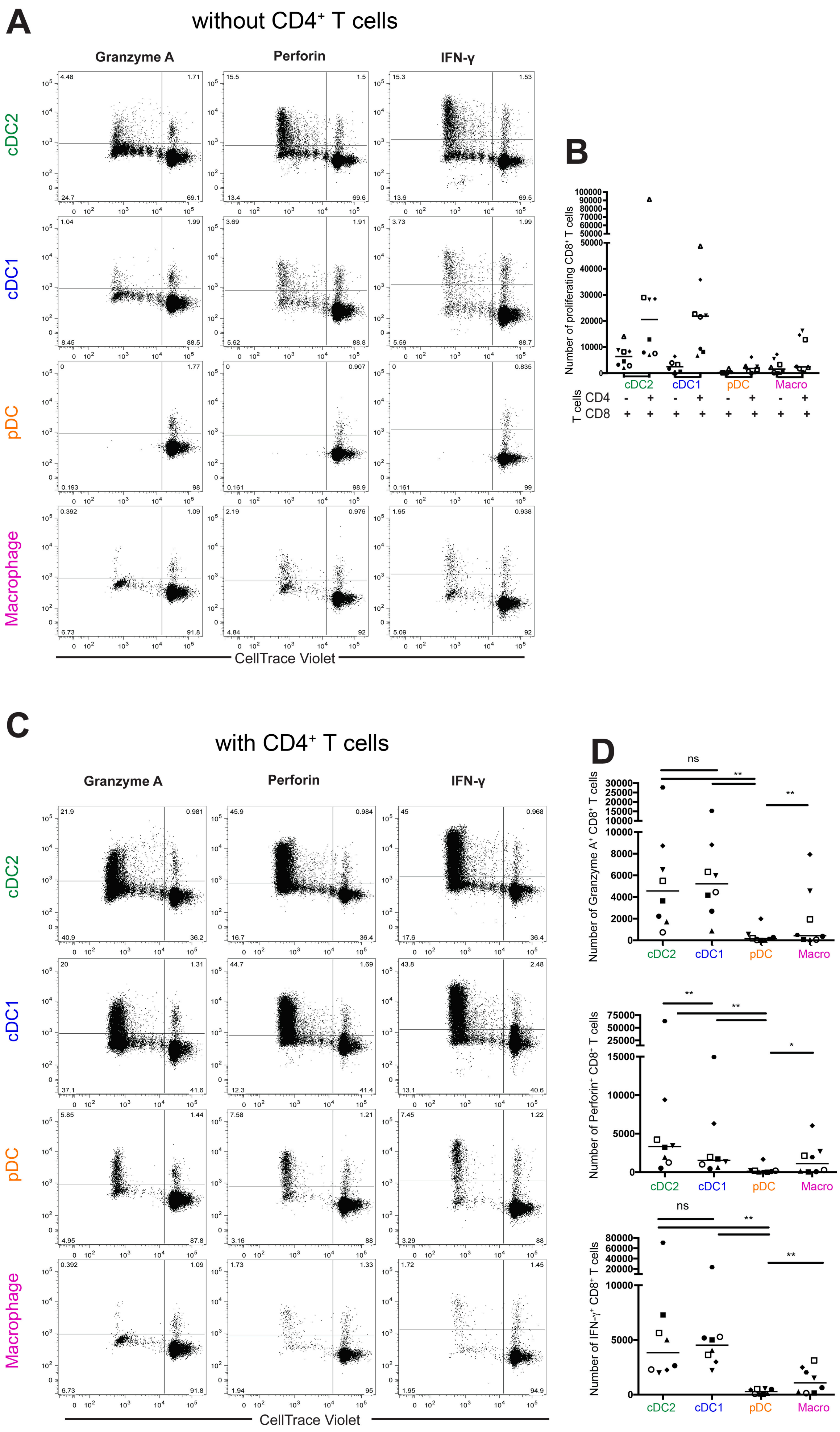
A
without CD4+ T cells
Granzyme A
Perforin
IFN-γ
cDC2
cDC1
pDC
Macrophage
CellTrace Violet
B
Number of proliferating CD8+ T cells
cDC2
cDC1
pDC
Macro
T cells
CD4
CD8
C
with CD4+ T cells
Granzyme A
Perforin
IFN-γ
cDC2
cDC1
pDC
Macrophage
CellTrace Violet
D
Number of Granzyme A+ CD8+ T cells
Number of Perforin+ CD8+ T cells
Number of IFN-γ+ CD8+ T cells
ns
**
*
cDC2
cDC1
pDC
Macro

Supplementary Figure 7. Induction of effector CD8$^+$ T cells by tonsil DC subsets. Purified DC and macrophage from tonsils were cultured with allogeneic CellTrace Violet-stained naïve CD8$^+$ T cells for 7 days, in the absence (A-B) or presence (C-D) of naïve CD4$^+$ T cells autologous to CD8$^+$ T cells. Expression of Granzyme A, Perforin and IFN-γ was assessed by intracellular flow cytometry. (A and C) Representative results of eight independent experiments. Gated on live CD8$^+$ T cells. (B) Number of proliferating CD8$^+$ T cells is shown. Symbols represent individual donors. Median is shown. (D) Number of CD8$^+$ T cells expressing effector molecules is shown. Symbols represent individual donors. N=8. Median is shown. * $p < 0.05$, ** $p < 0.01$, Wilcoxon non-parametric test.

Supplementary Methods

Clustree analysis. Clustree analysis was performed using the clustree package in R [1]. Results from clustering using Seurat package were then used to build a graph with resolution parameters ranging from 0 to 1.4, in steps of 0.1.

Supplementary References

1. Zappia L, Oshlack A. Clustering trees: a visualisation for evaluating clusterings at multiple resolutions. *Preprint at http://biorxiv.org* doi.org/10.1101/274035 (2018).
