# Supplementary material for: Human in vivo-generated monocyte-derived dendritic cells and macrophages cross-present antigens through a vacuolar pathway
Source: Nat Commun. 2018 Jul 2;9:2570. doi: 10.1038/s41467-018-04985-0 (PMC6028641; doi:10.1038/s41467-018-04985-0)
Supplement: Supplementary file 3 — Description of Additional Supplementary Files [file 41467_2018_4985_MOESM3_ESM.pdf]

Description of Additional Supplementary Files

File Name: Supplementary Data 1

Description: Gene lists for selected gene signatures
