# Supplementary material for: Human in vivo-generated monocyte-derived dendritic cells and macrophages cross-present antigens through a vacuolar pathway
Source: Nat Commun. 2018 Jul 2;9:2570. doi: 10.1038/s41467-018-04985-0 (PMC6028641; doi:10.1038/s41467-018-04985-0)
Supplement: Supplementary file 2 — Peer Review Report [file 41467_2018_4985_MOESM2_ESM.pdf]

Reviewers' comments:

Reviewer #1 (T-APC interaction, antigen presentation)(Remarks to the Author):

This paper examines the difficult issue of whether monocyte-derived DC and monocyte-derived macrophages recovered directly from humans can cross-present and, if so, by which pathway. This issue is of general interest, but is not well addressed by the study. The first two figures attempt to characterize the DC and macrophages within human ascites as monocyte-derived populations. Single cell RNA-seq data from ascites-derived cells isolated as DC or macrophages based on the surface markers (DR, CD11, CD1c and CD16), and from cells identified as blood-derived monocytes, is used to establish a number of different cell clusters within DC and Mac populations. These data are then used to establish single cell developmental trajectories shown as a pseudotime differentiation scheme outlined in figure 2a. While these data establish that some of the DC and Mac clusters in figure 1 may represent developmental stages along a differentiation pathway towards mo-DC or mo-Macs, the data do not establish that all DC and Mac in the samples are actually derived from monocytes. I think that if you for example included data from a non-mo DC in this analysis, it would likely fall at the end of branch B. This means that any function being assessed (for these DC and Mac) could be the consequence of contaminating DC that are not of monocyte origin. These analyses also imply that some of the cells within the Mac population (i.e. Mac3) could be precursors of DC. This second point means that Mac samples (which contain Mac1, Mac2 and Mac3 clusters) analysed for cross-presentation could be providing function through newly arising DC derived from Mac3 during culture. Thus, while the bioinformatic analysis of single cell RNA-seq data is interesting, it is not definitive and it does not convince this reviewer that the populations being identified and tested are monocyte-derived.

The authors show that ascites-derived populations designated as mo-DC and mo-Mac can cross-present and that they do not use the cytosolic pathway as cross-presentation is not inhibitable by a proteasome inhibitor. They then imply participation of the vacuolar pathway in cross-presentation by showing a pan-cathepsin inhibitor can block presentation. Unfortunately, because cathepsins are important for various cellular processes, this conclusion is questionable, as disrupting cathepsin function could simply affect the architecture of the endosomes, preventing correct trafficking of essential molecules such as MHC I. This indirect effect could alter any presentation pathway. For example, antigens may be being degraded in the supernatant of the culture, but cannot be presented because there is limited MHC I available. In fact, the partial reduction in the very efficient presentation of short peptide supports this view. One experiment to improve this piece of work would be to show that the cathepsin inhibitors do not inhibit cytosolic-mediated cross-presentation in control DC populations that use the cytosolic but not vascuolar pathway. It would also be useful to reduce the short peptide dose to give similar responses to the long peptide and then examine the effects of the cathepsin inhibitor.

Minor point page 5, second line. Should “designed” be “designated”?

Reviewer #2 (Antigen presentation; MHC)(Remarks to the Author):

Manuscript Nr: NCOMMS-17-33191
Tang-Huau et al., “Human naturally-occuring monocyte-derived dendritic cells cross-present antigens exclusively through a vacuolar pathway”

The authors demonstrate that ascites has both monocyte-derived myeloid populations with transcriptional hallmarks of monocyte-derived macrophages (mo-Mac) and DCs (moDCs). Surprisingly, the authors further found that mo-Macs cross-presented long peptides more

efficiently than mo-DCs. This cross-presentation was not accompanied with endosomal leakage or sensitive to proteasome inhibition. Instead pan-cathepsin inhibition compromised this cross-presentation. Only mo-DCs were, however, able to prime naïve allogeneic CD8+ T cells, but with similar efficacy as tonsillar DC1 and DC2 subsets. From these data the authors conclude that monocyte-derived inflammatory human DCs and macrophages use the vacuolar pathway and lysosomal degradation for antigen cross-presentation onto MHC class I molecules.

These are interesting findings, but further blur the difference between macrophages and DCs with respect to antigen presentation.

Major comments:
1. In their alignment of ascites derived myeloid populations it is very interesting that the authors find two divergent differentiation pathways towards monocyte-derived macrophages and DCs. However, it remains unclear if one DC or macrophage subset would cluster unsupervised with the transcriptome of in vitro derived mo-DC and mo-Mac without preselection of a mo-DC transcription signature. Have the authors done such principle component analyses? What was the outcome? What are the main GO terms distinguishing the two branches?
2. Is there any evidence in the transcription profiles that mo-Macs or mo-DCs have elevated lysosomal degradation capacities? It was previously suggested that an attenuated lysosomal compartment could be beneficial for cross-presentation. Do ascites derived mo-Macs have less lysosomal hydrolases, like cathepsins, than mo-DCs?
3. Long peptides are not the most physiological source of cross-presented antigens. Are other antigen formulations apart from long peptides, like cellular debris after apoptosis and/or necrosis also cross-presented by ascites derived mo-Macs and mo-DCs in a manner dependent on lysosomal proteolysis? Are mo-Macs also in this case more efficient in cross-presentation?
4. Despite this efficient cross-presentation, mo-Macs prime CD8+ T cell responses less efficiently. Why is this the case? Do the authors observe a co-stimulatory and/or cytokine signature at the transcription level that could explain the improved priming by mo-DCs versus mo-Macs?
5. The authors speculate that differences in co-stimulatory molecules (signal 2) and/or cytokines (signal 3) might explain the superior CD8+ T cell priming capacity of mo-DCs. Can they verify some of the respective changes like possibly CD70 and IL-12p35 at the protein level and functionally by blocking their contribution to CD8+ T cell priming?

Minor comments:
1. The authors emphasize in their manuscript title mo-DCs, but the more surprising finding is probably that mo-Macs cross-present antigen on MHC class I at least as efficiently. Therefore, it would be appropriate to extend the title to “naturally-occurring monocyte-derived dendritic cells and macrophages”.

In summary, these are interesting findings on a very interesting human source of monocyte-derived inflammatory myeloid cell populations. Some more information on their similarities and differences in the three signals for CD8+ T cell priming should be provided.

Reviewer #3 (MHC/TCR repertoire, antigen presentation)(Remarks to the Author):

This paper is an examination of monocyte derived dendritic cells and macrophages for their capacity to “cross present”, that is, the presentation of MHC-I epitopes to CD8 T cells. The paper has the merit that it examines human cells obtained from ascites, ie from the peritoneal cavity. The issue that is examined is whether the presenting cells use a pathway in which the proteins migrate to the cytosol or one that strictly centers on endocytic vesicles. This issue has been well discussed by many now, much depending on the nature of the immunogen and , or, the presenting cell, ie macrophages, subsets of DC, cell lines. The data that is presented in this study has many

flaws many of them technical, that need correction and reevaluations. A number of experiments are not critically evaluated and require more detail analysis.

The gene analysis is not convincing. Among several major concern are: i) monocytes on Fig1A-B are coming from donors B and C, while DCs and macrophages – exclusively from donor A. This will result in strong donor/batch effect, since the conditions and batches cannot be resolved; this represents a serious flaw of this analysis, that is unacceptable. The expression of selected genes was dependent on the sample origin of these clusters (fig.1C). – How were they selected? What are the top genes by p-value, log fold change? Note the results of Fig 1C showing a large overlap in expressions of key signature genes. In Fig1D-E: the main text states that “Clusters Mac1 and Mac2 had the highest expression of mo-Mac signature, while clusters DC2 and DC3 had the highest expression of mo-DC signature” But from the figure legends: “For each cell, the number of detected genes from the mo-Mac (D) or mo-DC (E) signature is depicted” – Please show not the number, but expression value. Other smaller issues are the removal of some clusters, such as those containing small number of cells or the cluster containing B cells.

In essence, the conclusion that “Overall, these results show that ascites DC and ascites macrophages are distinct populations, and comprise groups of cells with slightly different gene expression patterns.” is not warranted by the data as presented.

The exact steps taken to separate and purify DC from macrophages isolated from ascites is not spelled with the detail that it should.This is key information.

Fig 4 analyzes the transfer of beta lactamase to the cytosol. How is the assay controlled for the number of cells examined and to the input amount of the enzyme? Certainly in the panel A of Fig 4 ascites there is transfer from the ascites macrophages. And in panel C there is transfer close to 10% of cells in some of the assay, even including the DC. In brief there is concern on technical aspects, and how the results are interpreted. A more critical analysis is warranted.

Figure 5 shows a number of experiments, using proteasome and vacuolar inhibitors. But no evaluation is made of the macrophages. This should be done. In all different experiments the comparison is made of both cells except in this important one.

Finally a comparison is made of the function of the two cells to differentiate CD8 T cells to effector. Here a different assay employing allogeneic cells is used in which the function of CD4 is required but not explained. It appears that with this assay, the Dc are more effective. Why was the CD8 clones used in the cross presentation not examined in detail in its response to macrophages and DC, ie proliferation, differentiation etc rather than now using a different assay which adds a level of complexity and lacks clarity? I am not convinced that the absolute statement that “only ascites mp-DC induced cytotoxic CD8+ T cells” is warranted by the limited data that is presented.

Reply to reviewers' comments

Reviewer 1

1. The first two figures attempt to characterize the DC and macrophages within human ascites as monocyte-derived populations. Single cell RNA-seq data from ascites-derived cells isolated as DC or macrophages based on the surface markers (DR, CD11, CD1c and CD16), and from cells identified as blood-derived monocytes, is used to establish a number of different cell clusters within DC and Mac populations. These data are then used to establish single cell developmental trajectories shown as a pseudotime differentiation scheme outlined in figure 2a. While these data establish that some of the DC and Mac clusters in figure 1 may represent developmental stages along a differentiation pathway towards mo-DC or mo-Macs, the data do not establish that all DC and Mac in the samples are actually derived from monocytes. I think that if you for example included data from a non-mo DC in this analysis, it would likely fall at the end of branch B. This means that any function being assessed (for these DC and Mac) could be the consequence of contaminating DC that are not of monocyte origin. These analyses also imply that some of the cells within the Mac population (i.e. Mac3) could be precursors of DC. This second point means that Mac samples (which contain Mac1, Mac2 and Mac3 clusters) analysed for cross-presentation could be providing function through newly arising DC derived from Mac3 during culture. Thus, while the bioinformatic analysis of single cell RNA-seq data is interesting, it is not definitive and it does not convince this reviewer that the populations being identified and tested are monocyte-derived.

In the revised version of our manuscript, we aimed at improving the single-cell RNA-seq analysis. To increase the power of the analysis, we have now included single-cell RNA-seq data of cDC (lineage-negative HLA-DR^{+}CD11c^{+}CD14^{-} cells) purified from tonsil, to enable a direct comparison with ascites DC. We found that ascites DC and tonsil DC are not grouped in the same clusters, independently of the resolution used (see figure below). We agree with the reviewer that trajectory reconstruction is probably not the most relevant analysis to address whether ascites DC are monocyte-derived cells. Instead, we have now analysed in more depth differentially expressed genes (fig 1C and fig S1) and calculated signature scores for a number of signatures, from the literature (CD14+ monocytes, blood cDC1, blood cDC2, skin CD14+ cells) or that we designed (tissue cDC2, in vitro mo-Mac, in vitro mo-DC, activated DC) (fig 2 and table S1). While ascites DC and cDC2 share some of their transcriptional program, we also evidenced differential gene expression related to their ontogeny (fig S2). This data supports our conclusion that ascites DC do not contain a population of classical DC.

As for the possibility that mo-DC arise from the macrophage population during culture which would explain the cross-presentation results, we believe it is extremely unlikely. Indeed, in our cross-presentation assays, purified ascites cells are cultured for 3h with antigen in Yssel medium (i.e. not containing any growth factor or cytokine that may induce DC differentiation), washed then cultured overnight with the CD8 T cell clone, in Yssel medium with FCS (again not containing any growth factor or cytokine that may induce DC differentiation). Even if precursors of DC are present in the macrophage population, we think these cells would not have the time to

differentiate *in vitro* into mo-DC and to perform cross-presentation before the endpoint of the assay.

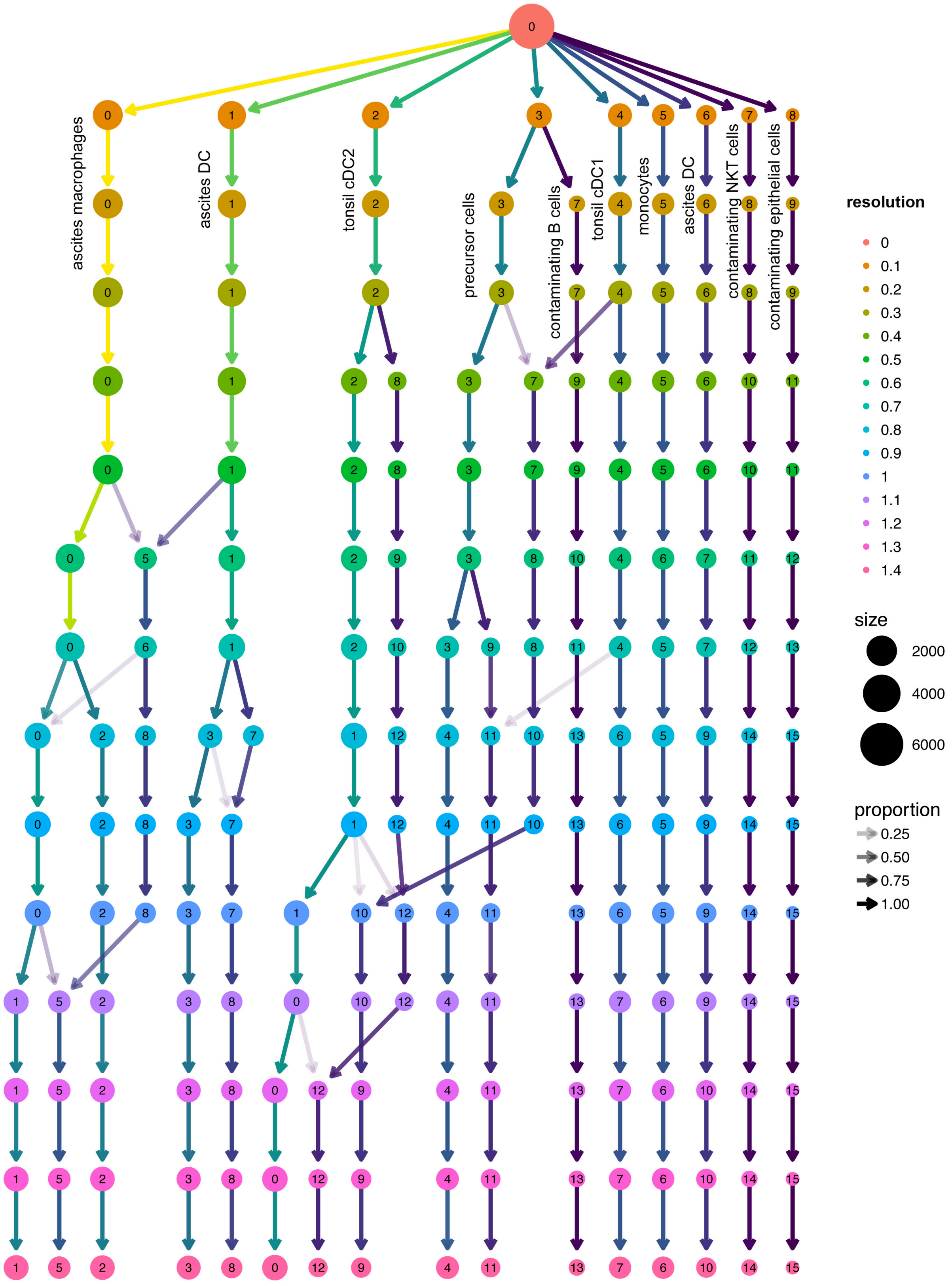

<u>Clustree analysis of the complete single-cell RNA-seq dataset</u>. Results from clustering using the Seurat package, with resolution parameters from 0 to 1.4. In this analysis, clusters are numbered based on the number of cells they contain. The 10 main branches that form starting at resolution 0.2 have been annotated for cell identity. Reference: Zappia and Oshlack (2018), doi.org/10.1101/274035.

2. The authors show that ascites-derived populations designated as mo-DC and mo-Mac can cross-present and that they do not use the cytosolic pathway as cross-presentation is not inhibitable by a proteasome inhibitor. They then imply participation of the vacuolar pathway in cross-presentation by showing a pan-cathepsin inhibitor can block presentation. Unfortunately, because cathepsins are important for various cellular processes, this conclusion is questionable, as disrupting cathepsin function could simply affect the architecture of the endosomes, preventing correct trafficking of essential molecules such as MHC I. This indirect effect could alter any presentation pathway. For example, antigens may be being degraded in the supernatant of the culture, but cannot be presented because there is limited MHC I available. In fact, the partial reduction in the very efficient presentation of short peptide supports this view. One experiment to improve this piece of work would be to show that the cathepsin inhibitors do not inhibit cytosolic-mediated cross-presentation in control DC populations that use the cytosolic but not vascuolar pathway. It would also be useful to reduce the short peptide dose to give similar responses to the long peptide and then examine the effects of the cathepsin inhibitor.

We thank the reviewer for these suggestions. The partial reduction in the presentation of the short peptide in the presence of cathepsin inhibitor was most likely due to the high concentration of DMSO introduced in this condition. Therefore, we repeated this experiment using the same concentration of DMSO as control condition (fig 5 C-F). We found that cross-presentation by both mo-DC and mo-Mac is impaired in the presence of the cathepsin inhibitor compared to DMSO alone (fig 5 D-E). In addition, cross-presentation by $CD1a^{+}$ DC was not affected by this inihibitor, while it was inhibited by lactacystin (fig 5C and 5F). Moreover, cell viability was similar with or without inhibitors (fig S5B). We believe that this new piece of data strengthens our conclusion that mo-DC and mo-Mac use the vacuolar pathway for cross-presentation.

Reviewer 2

1. In their alignment of ascites derived myeloid populations it is very interesting that the authors find two divergent differentiation pathways towards monocyte-derived macrophages and DCs. However, it remains unclear if one DC or macrophage subset would cluster unsupervised with the transcriptome of in vitro derived mo-DC and mo-Mac without preselection of a mo-DC transcription signature. Have the authors done such principle component analyses? What was the outcome? What are the main GO terms distinguishing the two branches?

In the revised version of our manuscript, we aimed at improving the single-cell RNA-seq analysis. To increase the power of the analysis, we have now included single-cell RNA-seq data of cDC purified from tonsil, to enable a direct comparison with ascites DC. Principal Component Analysis was performed on normalized gene-barcode matrix. Then we performed unsupervised clustering using the first 19 principal components. We found that ascites DC and tonsil DC are not grouped in the same clusters, independently of the resolution used (see figure above).

We have decided not to apply trajectory reconstruction on this data set, as it did not appear to us as the most relevant analysis. Instead, we have now analysed in more depth differentially expressed genes (fig 1C and fig S1) and calculated signature scores for a number of signatures, from the literature (CD14+ monocytes, blood cDC1, blood cDC2, skin CD14+ cells) or that we designed (tissue cDC2, in vitro mo-Mac, in vitro mo-DC, activated DC) (fig 2 and table S1). While ascites DC and cDC2 share some of their transcriptional program, we also evidenced differential gene expression related to their ontogeny (fig S2). This data supports our conclusion that ascites DC do not contain a population of classical DC.

2. Is there any evidence in the transcription profiles that mo-Macs or mo-DCs have elevated lysosomal degradation capacities? It was previously suggested that an attenuated lysosomal compartment could be beneficial for cross-presentation. Do ascites derived mo-Macs have less lysosomal hydrolases, like cathepsins, than mo-DCs?

Transcriptomic analysis showed that mo-Mac express higher levels of lysosomal proteases than mo-DC (fig S5A). However, it is difficult to draw conclusions from this analysis regarding their respective degradation capacities. To address whether there is evidence at the transcriptomic level for a differential ability for antigen presentation between ascites mo-DC and mo-Mac, we performed Gene Set Enrichment Analysis (fig S3A). We did not find any enrichment in either population for the expression of gene signatures related to antigen presentation, consistent with the results from the cross-presentation assay.

3. Long peptides are not the most physiological source of cross-presented antigens. Are other antigen formulations apart from long peptides, like cellular debris after apoptosis and/or necrosis also cross-presented by ascites derived mo-Macs and mo-DCs in a manner dependent on lysosomal proteolysis? Are mo-Macs also in this case more efficient in cross-presentation?

We have now performed cross-presentation using MelanA-coated beads as a model for particulate antigen (fig 3C). We found that both mo-DC and mo-Mac could efficiently cross-present this form of antigen. We also show that mo-Mac express higher levels of HLA-A2 (fig S3C), which could explain their superior ability for stimulation of the CD8 T cell clone with the short peptide and for cross-presentation.

4. Despite this efficient cross-presentation, mo-Macs prime CD8+ T cell responses less efficiently. Why is this the case? Do the authors observe a co-stimulatory and/or cytokine signature at the transcription level that could explain the improved priming by mo-DCs versus mo-Macs?

We have addressed this question in new figure 7. We found that ascites mo-DC express higher levels of co-stimulatory molecules than ascites mo-Mac (fig 7A). In our transcriptomics dataset, we do not observe strong expression for a number of cytokines, including IL12, probably because the cells need to be stimulated in order to express the mRNA for such cytokines.

5. The authors speculate that differences in co-stimulatory molecules (signal 2) and/or cytokines (signal 3) might explain the superior CD8+ T cell priming capacity of

mo-DCs. Can they verify some of the respective changes like possibly CD70 and IL-12p35 at the protein level and functionally by blocking their contribution to CD8+ T cell priming?

We have addressed this question in new figure 7. We show that in our co-culture assay with naïve T cells, ascites mo-DC are more efficient for inducing naïve CD4 T cell proliferation (fig 7B), thereby potentially providing more help for the differentiation of cytotoxic CD8 T cells. We also show that only ascites mo-DC, but not mo-Mac, secrete IL12p70 upon *ex vivo* stimulation (fig 7C). Collectively, these results indicate that only ascites mo-DC can provide the co-stimulatory signals required for efficient differentiation of effector cytotoxic CD8 T cells.

Reviewer 3

1. The gene analysis is not convincing. Among several major concern are: i) monocytes on Fig1A-B are coming from donors B and C, while DCs and macrophages – exclusively from donor A. This will result in strong donor/batch effect, since the conditions and batches cannot be resolved; this represents a serious flaw of this analysis, that is unacceptable.

In the revised version of our manuscript, we aimed at improving the single-cell RNA-seq analysis. To increase the power of the analysis, we have now included single-cell RNA-seq data of cDC purified from tonsil, to enable a direct comparison with ascites DC. There is no consensus in the field as to whether strong batch or donor effects exist with this type of data. To mitigate for batch effects, we have performed regression in gene expression based on the number of unique molecular identifiers and the percentage of mitochondrial genes. Our dataset provides some internal control: 1) monocytes coming from 2 differents donors are grouped in the same cluster; 2) cells with similar transcriptional profiles (cell cycle genes) were grouped in the same cluster independently of their sample origin (fig 1A-B). Therefore, we believe that our analysis is valid.

2. The expression of selected genes was dependent on the sample origin of these clusters (fig.1C). – How were they selected? What are the top genes by p-value, log fold change? Note the results of Fig 1C showing a large overlap in expressions of key signature genes.

We have modified figure 1 for a better visualisation of the single-cell RNA-seq results. We now show the top differentially expressed genes for each cluster (based on log fold change) as a heatmap in fig 1C, and as violin plots in fig S1.

3. In Fig1D-E: the main text states that “Clusters Mac1 and Mac2 had the highest expression of mo-Mac signature, while clusters DC2 and DC3 had the highest expression of mo-DC signature” But from the figure legends: “For each cell, the number of detected genes from the mo-Mac (D) or mo-DC (E) signature is depicted” – Please show not the number, but expression value.

We have now performed a more extensive analysis of the expression of gene signatures (fig 2). We have calculated signature scores for a number of signatures,

from the literature (CD14+ monocytes, blood cDC1, blood cDC2, skin CD14+ cells) or that we designed (tissue cDC2, in vitro mo-Mac, in vitro mo-DC, activated DC) (fig 2 and table S1). We believe that this new analysis makes the interpretation of the single-cell RNA-seq data easier.

4. Other smaller issues are the removal of some clusters, such as those containing small number of cells or the cluster containing B cells.

We now include in the 'material and methods' section more details about the cells that were excluded from the analysis (including number of cells and top differentially expressed genes). These populations were inflammatory B cells from ascites, NK T cells from tonsil and epithelial cells from both ascites and tonsil. For the reviewer's appreciation is below a hierarchical clustering analysis of the total dataset. This analysis clearly shows that the 3 contaminating populations are unrelated to the mononuclear phagocyte populations. Therefore, we believe it is not an issue to remove these cells from the subsequent analysis.

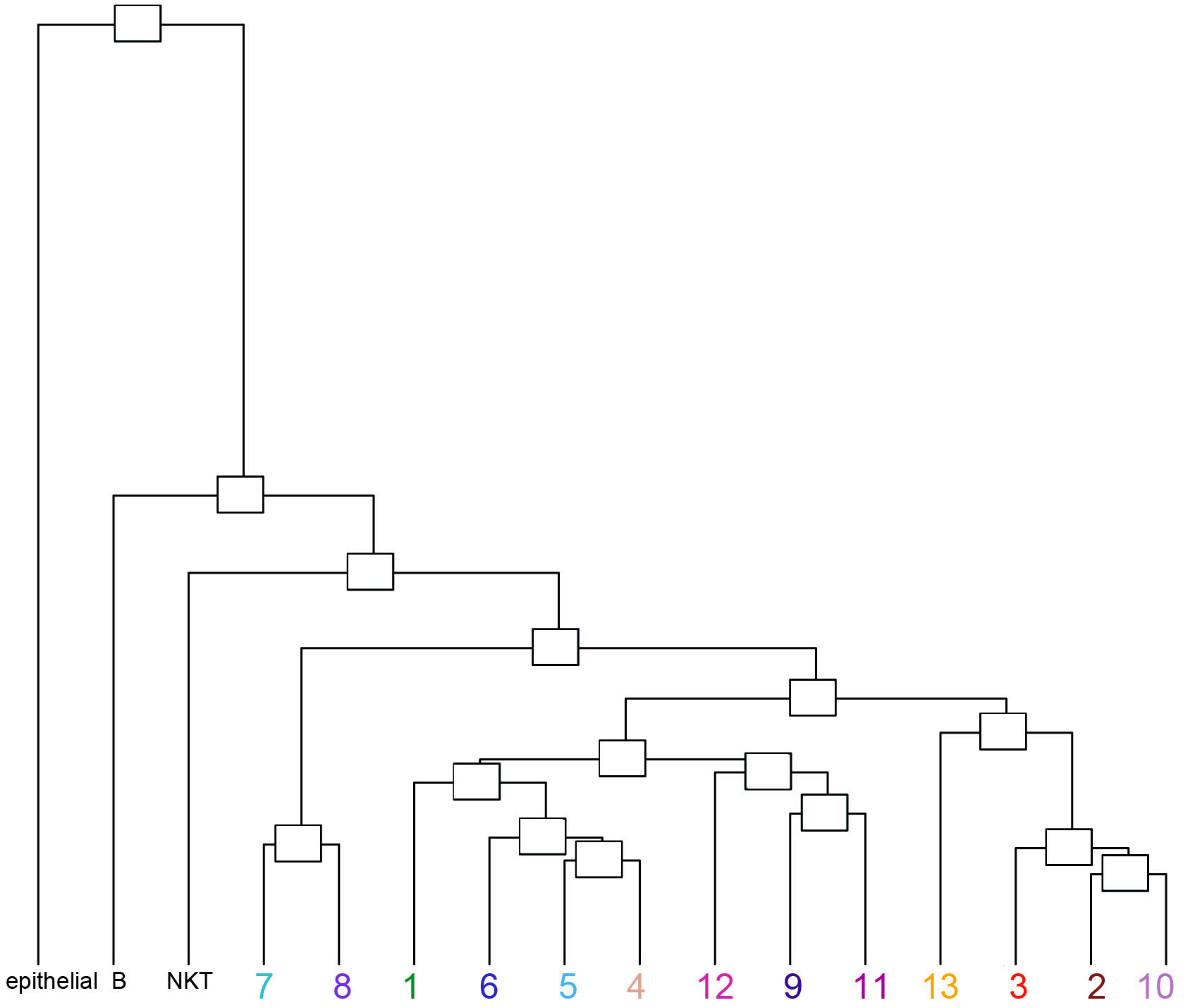

<u>Hierarchical clustering tree</u>. We constructed a phylogenetic tree relating the 'average' cell from each identity class. Tree is estimated based on a distance matrix constructed in PCA space using the same 19 principal components as for the graph-based clustering shown in fig 1. Corresponding clusters are indicated with their color code.

5. The exact steps taken to separate and purify DC from macrophages isolated from ascites is not spelled with the detail that it should.This is key information.

We have now completed the ‘material and methods’ section to include all relevant information.

6. Fig 4 analyzes the transfer of beta lactamase to the cytosol. How is the assay controlled for the number of cells examined and to the input amount of the enzyme? Certainly in the panel A of Fig 4 ascites there is transfer from the ascites macrophages. And in panel C there is transfer close to 10% of cells in some of the assay, even including the DC. In brief there is concern on technical aspects, and how the results are interpreted. A more critical analysis is warranted.

We have now included more details in the ‘material and methods’ section for the beta-lactamase assay. The number of cells and the concentration of beta-lactamase are standardized. In addition, the internalisation assay with fluorescent beta-lactamase (fig 4C) shows that the amount of internalized beta-lactamase is similar between mo-DC and mo-Mac. We have also modified the wording of our interpretation of these results (page 9) and the title of the figure (fig 4).

7. Figure 5 shows a number of experiments, using proteasome and vacuolar inhibitors. But no evaluation is made of the macrophages. This should be done. In all different experiments the comparison is made of both cells except in this important one.

We have now included mo-Mac in these experiments (fig 5). We found that, similar to mo-DC, cross-presentation by mo-Mac is inhibited by the pan-cathepsin inhibitor, but not by the proteasome inhibitor lactacystin (fig 5B and 5E). In addition, we confirmed that proteasome activity was indeed inhibited by lactacystin in both mo-DC and mo-Mac at the concentration used in our cross-presentation assay (fig S4).

8. Finally a comparison is made of the function of the two cells to differentiate CD8 T cells to effector. Here a different assay employing allogeneic cells is used in which the function of CD4 is required but not explained. It appears that with this assay, the Dc are more effective. Why was the CD8 clones used in the cross presentation not examined in detail in its response to macrophages and DC, ie proliferation, differentiation etc rather than now using a different assay which adds a level of complexity and lacks clarity? I am not convinced that the absolute statement that “only ascites mp-DC induced cytotoxic CD8+ T cells” is warranted by the limited data that is presented.

The CD8 T cell clone that we used in our cross-presentation assay does not proliferate and does not differentiate into cytotoxic T cells. To address this question, we had to turn to a different culture model. It was previously shown in the literature that differentiation of cytotoxic CD8 T cells requires help from CD4 T cells, both *in vivo* in the mouse and *in vitro* in human culture systems. We confirmed this observation in our assay. We have modified the text to better explain this point.
To improve this part of the manuscript, we also analysed signals required for effector CD8 T cell differentiation. We found that ascites mo-DC express higher levels of co-stimulatory molecules than ascites mo-Mac (fig 7A). In our co-culture assay with

naïve T cells, ascites mo-DC are more efficient for inducing naïve CD4 T cell proliferation (fig 7B), thereby potentially providing more help for the differentiation of cytotoxic CD8 T cells. Finally, we show that only ascites mo-DC, but not mo-Mac secrete IL12p70 (fig 7C). Collectively, these results show that only ascites mo-DC can provide the co-stimulatory signals required for efficient differentiation of effector cytotoxic CD8 T cells.

REVIEWERS' COMMENTS:

Reviewer #1 (Remarks to the Author):

The authors have adequately addressed all of my concerns.

Reviewer #2 (Remarks to the Author):

Manuscript Nr: NCOMMS-17-33191A
Tang-Huau et al., “Human naturally-occuring monocyte-derived dendritic cells cross-present antigens exclusively through a vacuolar pathway”

The authors demonstrate that ascites has both monocyte-derived myeloid populations with transcriptional hallmarks of monocyte-derived macrophages (mo-Mac) and DCs (moDCs). Surprisingly, the authors further found that mo-Macs cross-presented long peptides more efficiently than mo-DCs. This cross-presentation was not accompanied with endosomal leakage or sensitive to proteasome inhibition. Instead pan-cathepsin inhibition compromised this cross-presentation. Only mo-DCs were, however, able to prime naïve allogeneic CD8+ T cells, but with similar efficacy as tonsillar DC1 and DC2 subsets. From these data the authors conclude that monocyte-derived inflammatory human DCs and macrophages use the vacuolar pathway and lysosomal degradation for antigen cross-presentation onto MHC class I molecules.

In their revised manuscript version the authors have addressed most of my concerns, including additional analysis of the transcriptome profiles, mining them for antigen processing pathways, cross-presenting assays for particulate antigen (antigen loaded beads) and providing additional data on the mechanistic basis of the superior T cell priming capacity of human monocyte-derived DCs. These additional data have significantly improved the manuscript, but the authors still seem to hide the surprising cross-presentation capacity of monocyte-derive macrophages, even so their assays clearly document it.

Minor comments:
1. The authors emphasize in their manuscript title mo-DCs, but the more surprising finding is probably that mo-Macs cross-present antigen on MHC class I at least as efficiently. Therefore, it would be appropriate to extend the title to “naturally-occurring monocyte-derived dendritic cells and macrophages”.

In summary, these are interesting findings on a very interesting human source of monocyte-derived inflammatory myeloid cell populations.

Reviewer #3 (Remarks to the Author):

The authors have made an effort to answer all the various issues that were raised. Most of the major points that I raised as reviewer 3 have been answered to my satisfaction. The data supports the major statements and conclusions. I also believe that the other major points have been dealt adequately.

REPLY TO REVIEWERS' COMMENTS

Reviewer #2 (Remarks to the Author):

Manuscript Nr: NCOMMS-17-33191A
Tang-Huau et al., “Human naturally-occuring monocyte-derived dendritic cells cross-present antigens exclusively through a vacuolar pathway”

The authors demonstrate that ascites has both monocyte-derived myeloid populations with transcriptional hallmarks of monocyte-derived macrophages (mo-Mac) and DCs (moDCs). Surprisingly, the authors further found that mo-Macs cross-presented long peptides more efficiently than mo-DCs. This cross-presentation was not accompanied with endosomal leakage or sensitive to proteasome inhibition. Instead pan-cathepsin inhibition compromised this cross-presentation. Only mo-DCs were, however, able to prime naïve allogeneic CD8+ T cells, but with similar efficacy as tonsillar DC1 and DC2 subsets. From these data the authors conclude that monocyte-derived inflammatory human DCs and macrophages use the vacuolar pathway and lysosomal degradation for antigen cross-presentation onto MHC class I molecules.

In their revised manuscript version the authors have addressed most of my concerns, including additional analysis of the transcriptome profiles, mining them for antigen processing pathways, cross-presenting assays for particulate antigen (antigen loaded beads) and providing additional data on the mechanistic basis of the superior T cell priming capacity of human monocyte-derived DCs. These additional data have significantly improved the manuscript, but the authors still seem to hide the surprising cross-presentation capacity of monocyte-derive macrophages, even so their assays clearly document it.

Minor comments:
1. The authors emphasize in their manuscript title mo-DCs, but the more surprising finding is probably that mo-Macs cross-present antigen on MHC class I at least as efficiently. Therefore, it would be appropriate to extend the title to “naturally-occurring monocyte-derived dendritic cells and macrophages”.

In summary, these are interesting findings on a very interesting human source of monocyte-derived inflammatory myeloid cell populations.

To address the reviewer’s comments, we have modified the title of the manuscript to include the word ‘macrophages’. We have also added some discussion about the cross-presenting ability of macrophages.
